# Exome sequencing of families from Ghana reveals known and candidate hearing impairment genes

**DOI:** 10.1038/s42003-022-03326-8

**Published:** 2022-04-19

**Authors:** Ambroise Wonkam, Samuel Mawuli Adadey, Isabelle Schrauwen, Elvis Twumasi Aboagye, Edmond Wonkam-Tingang, Kevin Esoh, Kalinka Popel, Noluthando Manyisa, Mario Jonas, Carmen deKock, Victoria Nembaware, Diana M. Cornejo Sanchez, Thashi Bharadwaj, Abdul Nasir, Jenna L. Everard, Magda K. Kadlubowska, Liz M. Nouel-Saied, Anushree Acharya, Osbourne Quaye, Geoffrey K. Amedofu, Gordon A. Awandare, Suzanne M. Leal

**Affiliations:** 1 Division of Human Genetics, Faculty of Health Sciences, University of Cape Town, Cape Town, 7925 South Africa; 2 McKusick-Nathans Institute and Department of Genetic Medicine, Johns Hopkins University School of Medicine, Baltimore, MD 21205 USA; 3 West African Centre for Cell Biology of Infectious Pathogens (WACCBIP), University of Ghana, Accra, LG 54 Ghana; 4 Center for Statistical Genetics, Gertrude H. Sergievsky Center, and the Department of Neurology, Columbia University Medical Centre, New York, NY 10032 USA; 5 Department of Molecular Science and Technology, Ajou University, Suwon-si, Republic of Korea; 6 Department of Eye, Ear, Nose, and Throat, School of Medical Sciences, Kwame Nkrumah University of Science and Technology, Kumasi, Ghana; 7 Taub Institute for Alzheimer’s Disease and the Aging Brain, Columbia University Medical Centre, New York, NY 10032 USA

**Keywords:** Medical genetics, Molecular medicine

## Abstract

We investigated hearing impairment (HI) in 51 families from Ghana with at least two affected members that were negative for *GJB2* pathogenic variants. DNA samples from 184 family members underwent whole-exome sequencing (WES). Variants were found in 14 known non-syndromic HI (NSHI) genes [26/51 (51.0%) families], five genes that can underlie either syndromic HI or NSHI [13/51 (25.5%)], and one syndromic HI gene [1/51 (2.0%)]. Variants in *CDH23* and *MYO15A* contributed the most to HI [31.4% (16/51 families)]. For *DSPP*, an autosomal recessive mode of inheritance was detected. Post-lingual expression was observed for a family segregating a *MARVELD2* variant. To our knowledge, seven novel candidate HI genes were identified (13.7%), with six associated with NSHI (*INPP4B*, *CCDC141, MYO19, DNAH11, POTEI*, and *SOX9*); and one (*PAX8*) with Waardenburg syndrome. *MYO19* and *DNAH11* were replicated in unrelated Ghanaian probands. Six of the novel genes were expressed in mouse inner ear. It is known that *Pax8*^*-/-*^ mice do not respond to sound, and depletion of Sox9 resulted in defective vestibular structures and abnormal utricle development. Most variants (48/60; 80.0%) have not previously been associated with HI. Identifying seven candidate genes in this study emphasizes the potential of novel HI genes discovery in Africa.

## Abstract (web summary)

Whole-exome sequencing for 51 families in Ghana reveals 7 candidate genes associated with hearing impairment, 6 of which have previously been demonstrated to be expressed in the mouse inner ear.

## Introduction

Hearing impairment (HI) is a sensory disorder that can be mild to profound and has an incidence of 2–3 out of 1000 live births in developed countries and twice as high in developing countries^1^. HI is often caused by acquired factors such as infectious diseases and antibiotic exposure^2–4^. Genetic factors are known to cause ~50% of congenital HI^2,5^, and to date, >120 non-syndromic (NS) HI genes have been identified^6^. *GJB2* is the most common gene associated with autosomal recessive (AR) NSHI among European populations^7–9^.

Although connexin genes are the most widely reported HI genes, their contribution to HI in sub-Saharan African populations is negligible^10^. However, Ghana is an exception with a high frequency of the founder *GJB2-*p.R143W variant^3,11,12^, which accounts for at least 27% of HI in Ghanaian families segregating ARNSHI^13^, 8% of isolated NSHI cases, and a population carrier frequency of 0.7%^3^.

Considering the highly heterogeneous status of HI^6^, next-generation sequencing platforms and particularly whole-exome sequencing (WES) have recently enhanced the discovery of novel HI gene variants^14,15^. In the current study of families from Ghana, we used WES to investigate rare variants in *GJB2*-negative families segregating HI.

## Results

### Patients’ demographics and phenotypic descriptions

**Fig. 1:**
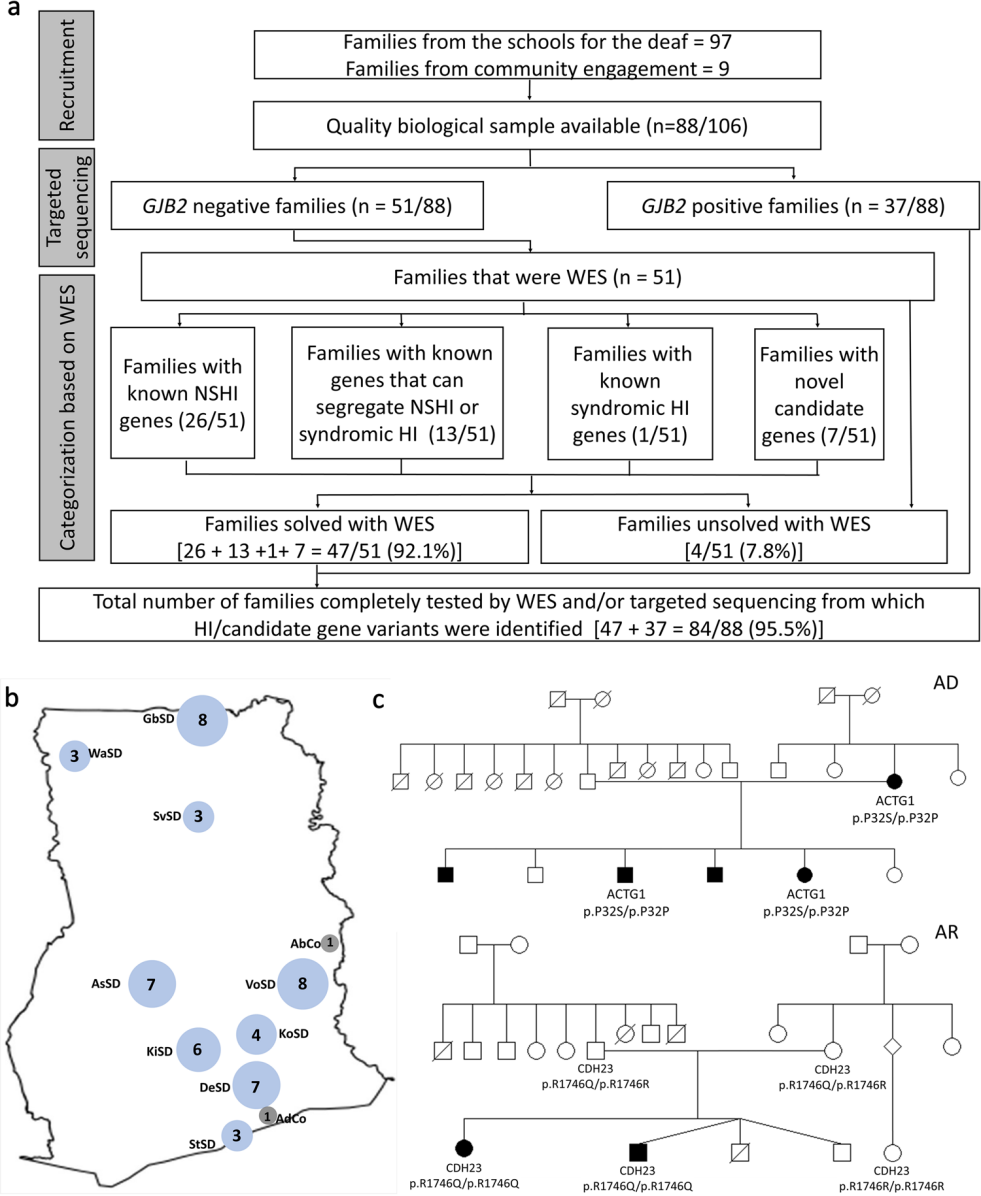
Summary of patient enrollment in Ghana. **a** Flow diagram of the study. **b** National recruitment sites for families with HI (*n* = 51) considered for WES. Blue circles denote schools for the deaf and the number of recruited families written in the circles. State School for the Deaf (StSD) in Greater Accra Region, Gbeogo School for the Deaf (GbSD) in Upper East Region, Volta School for the Deaf (VoSD) in Volta Region, Ashanti School for the Deaf (AsSD) in Ashanti Region, Koforidua School for the Deaf (KoSD), Kyebi School for the Deaf (KiSD), and Demonstration School for the Deaf (DeSD) in Eastern Region, Savelugu School for the Deaf (SvSD) in Northern Region, and Wa School for the Deaf (WaSD) in Upper West Region. Gray circles represent communities, Abotoase (AbCo), and Adamorobe (AdCo). **c** Representative families with autosomal dominant (AD) and autosomal recessive (AR) mode of inheritance.

The study participants were recruited across the country with the majority from the Eastern region of Ghana (Fig. 1a, b), which has the highest number of schools for the deaf. These are boarding schools with students mostly from the surrounding towns and villages. A total of 88 families were ascertained, of which 37 could be explained by *GJB2* mutations leaving 51 families for further study (Fig. 1a). The number of affected females and males included in the study (from whom WES was performed) who are negative for *GJB2* variants was 48 (46.2%) and 56 (53.8%) (Supplementary Fig. 1), respectively.

The analysis of the student records retrieved from the schools for the deaf showed that all the students had bilateral HI. For the 51 pedigrees for which exome sequence data were generated, 50 (98.0%) families have members with prelingual HI while one family (Fam18, Supplementary Fig. 1g) has affected members with post-lingual HI, that developed HI at >8 years of age. Except for the post-lingual family, none of the hearing-impaired participants have verbal communication and are only able to communicate through sign language.

Although the student records confirmed that all the students had sensorineural HI, we were only able to retrieve audiograms for 39 study participants. The audiogram for most of the participants could not be obtained from the schools’ archives, and several of them had written audiological reports without an attached audiogram from the hearing assessment center. For the retrieved audiograms, the hearing threshold across all frequencies tested was 101.8 dB (standard deviation = 10.3) in the better ear. There was no significant difference in the pure tone average of the left and right ears for the hearing-impaired participants. Two unrelated families 2/51 (3.9%) included in the study clinically expressed phenotypes of Waardenburg syndrome (Supplementary Fig. 1f).

### Pedigree analysis

The analysis of the families enrolled in the study showed that the majority [37/51, (72.5%)] families) exhibited a likely AR mode of inheritance (Fig. 1c, Tables 1–3 and Supplementary Figs. 2–6). Six families showed a pattern of inheritance compatible with an autosomal dominant (AD) mode of inheritance (11.8%) (Fig. 1d, Tables 1, 3 and Supplementary Figs. 2–6). One family (2.0%) had a possible X-linked recessive mode of inheritance. The mode of inheritance in seven families was inconclusive since the pattern of inheritance is compatible with AR and X-linked recessive. None of the families segregated HI in a fashion that is compatible with mitochondrial inheritance. The average number of family members for whom a DNA sample was obtained is four. The maximum number of affected individuals per family was five (*n* = 2 families), with most families having two affected members (*n* = 38 families) (Supplementary Fig. 1). WES data were generated on DNA samples obtained from one affected member for two families, two affected members for 45 families, and three affected members for four families (*n* = 104; Supplementary Fig. 1). From all families, a total of 9 affected and 31 unaffected participants had available DNA but were not exome sequenced.

**Table 1:** Ghanaian families with variants in known genes underlying non-syndromic hearing impairment.

Gene	F-ID	Nucleotide change	Protein change	Rs-number	Inh	GT	Other HI gene variants detected that do not segregate with HI
*MYO15A*	Fam9	c.4778A>G	p.(E1593G)	–	AR	Het	–
		c.6011C>T	p.(P2004L)	rs756172590	AR	Het	–
*MYO15A*	Fam11	c.8767C>T	p.(R2923X)	rs373462792	AR	Het	–
		c.6551G>C	p.(C2184S)	–	AR	Het	–
*MYO15A*	Fam13	c.8340G>A	p.(T2780T) (splicing)	rs878853228	AR	Het	*C1QBP*: c.241G>A: p.(A81T) (rs145848155) (Hom)
		c.4216G>A	p.(E1406K)	rs759810756	AR	Het	
*MYO15A*	Fam21	c.6716A>C	p.(H2239P)	rs760577812	AR	Het	*MYO15A:* c.1196A>G, p.(Y399C) (rs368682932); *GREB1L*: c.4840A>G: p.(I1614V) (rs1032115325) (Het)
		c.8065delT	p.(W2689Gfs*49)	rs1567654885	AR	Het	
*MYO15A*	Fam22	c.6518C>T	p.(S2173F)	–	AR	Het	*SRCAP*: c.2821A>T: p.(I941L) (rs1176169022) (Het)
		c.6863C>T	p.(S2288L)	rs886052676	AR	Het	–
*MYO15A*	Fam23	c.4519C>T	p.(R1507X)	rs549138385	AR	Het	–
		c.6011C>T	p.(P2004L)	rs756172590	AR	Het	–
*MYO15A*	Fam34	c.6302T>C	p.(L2101P)	rs201908493	AR	Het	–
		c.6634G>A	p.(E2212K)	rs371352836	AR	Het	–
*MYO15A*	Fam50	c.8340G>A	p.(T2780T) (splicing)	rs878853228	AR	Het	–
		c.9947A>G	p.(Q3316R)		AR	Het	–
*GIPC3*	Fam10	c.241C>T	p.(L81F)	rs1487341857	AR	Hom	–
*GIPC3*	Fam30	c.216_ 225del	p.(P73Ffs*21)	–	AR	Hom	–
*GIPC3*	Fam31	c.241C>T	p.(L81F)	rs1487341857	AR	Hom	–
*OTOF*	Fam32	c.1051C>T	p.(Q351X)	rs1558492758	AR	Hom	–
*OTOF*	Fam14	c.5813+1G>A			AR	Het	–
		c.1747C>G	p.(P583A)	rs756894987	AR	Het	–
		c.1479_ 1481del	p.(K493_ R494delinsN)	–	AR	Het	–
*OTOF*	Fam36	c.1479_ 1481del	p.(K493_ R494delinsN)	–	AR	Hom	*PCDH7*: c.3659A>G: p.(N1220S) (Hom)
*ESPN*	Fam1	c.2254_2275del+c.2279G>T^a^	p.(E731Cfs*6) + p.(R760L)	rs371306949	AR	Hom	–
*ESPN*	Fam3	c.2254_2275del+c.2279G>T^a^	p.(E731Cfs*6) + p.(R760L)	rs371306949	AR	Hom	–
*GRXCR1*	Fam5	c.784C>T	p.(R262X)	rs761349153	AR	Hom	–
*MARVELD2*	Fam18^b^	c.1058dupT	p.(V354Sfs*5)		AR	Hom	–
*KARS*	Fam19	c.1685G>C	p.(C562S)	rs1156833108	AR	Hom	–
*TRRAP*	Fam27	c.1156G>A	p.(V386I)	–	AD	Het	[*TMEM132E*: c.470G>C: p.(G157A) (rs151214976) (het)], [*SLC17A8*: c.904-6C>A (Het)]
*MYH14*	Fam28	c.1775G>A	p.(R592Q)	rs1445498283	AD	Het	*MYO7A*: c.2095-1G>A (Hom)
*OTOG*	Fam33	c.6982T>A	p.(S2328T)	rs1274985459	AR	Het	–
		c.8513G>A	p.(R2838H)	rs544815967	AR	Het	*TPRN*: c.107delG: p.(G36Afs*414) (rs1011757302) (Hom)
*DSPP*	Fam37	c.2306A>G	p.(D769G)	rs370931212	AR^c^	Het	*AHCY*: c.1075G>T: p.(V359L) (Het)
		c.2502_2518del	p.(D834Efs*2)	–	AR^c^	Het	–
*LOXHD1*	Fam38	c.3734G>A	p.(G1245D)	–	AR	Hom	–
		c.5050G>A	p.(A1684T)	rs376122149	AR	Hom	–
*TMC1*	Fam47	c.1622T>A	p.(I541N)	–	AR	Hom	–
*SLC12A2*	Fam35	c.2935G>A	p.(E979K)	rs1581138934	AD	Het	–

*Rs-number* reference number, *Inh* mode of inheritance, *hom* homozygote, *het* heterozygote, *GT* patient genotype.

^a^Variants are both on the same haplotype, the missense variant (R760L) is predicted to be at position 753 as part of the frameshift sequence.

^b^These families feature a HI with post-lingual expression, an expansion of the phenotype of *MARVELD2*.

^c^These variants are inherited via a different inheritance model than those known for this gene.

**Table 2:** Known genes associated with both non-syndromic and syndromic HI found in Ghanaian families.

Gene	F-ID	Nucleotide change	Protein change	Rs-number	Inh	GT	Other HI gene variants found in the listed families, but not segregating with HI	Syndrome
*Genes associated with both non-syndromic and syndromic HI*								
*CDH23*	Fam15	c.2206C>T	p.(R736X)	rs1230303971	AR	Hom		Usher syndrome, type 1D
*CDH23*	Fam7	c.5237G>A	p.(R1746Q)	rs111033270	AR	Hom		Usher syndrome, type 1D
*CDH23*	Fam12	c.2746G>A	p.(D916N)	rs1318444606	AR	Het		Usher syndrome, type 1D
		c.4562A>G	p.(N1521S)	rs780987516	AR	Het		Usher syndrome, type 1D
*CDH23*	Fam24	c.3181G>A	p.(E1061K)	rs1060499793	AR	Het		Usher syndrome, type 1D
		c.6514C>T	p.(P2172S)	–	AR	Het		Usher syndrome, type 1D
*CDH23*	Fam41	c.4562A>G	p.(N1521S)	rs780987516	AR	Hom		Usher syndrome, type 1D
*CDH23*	Fam46	c.2746G>A	p.(D916N)	rs1318444606	AR	Hom		Usher syndrome, type 1D
*CDH23*	Fam43	c.4562A>G	p.(N1521S)	rs780987516	AR	Hom		Usher syndrome, type 1D
*CDH23*	Fam29	c.2206C>T	p.(R736X)	rs1230303971	AR	Het		Usher syndrome, type 1D
		c.4562A>G	p.(N1521S)	rs780987516	AR	Het		Usher syndrome, type 1D
*MYO7A*	Fam8	c.1996C>T	p.(R666X)	rs121965085	AR	Het		Usher syndrome, type 1B
		c.5101C>T	p.(R1701X)	rs111033182	AR	Het		
*CIB2*	Fam42	c.556C>T	p.(R186W)	rs370359511	AR	Hom	[*CIB2*:c.409C>T: p.(R137W) (Hom)], [*CIB2*: c.427C>T: p.(R143W) (Hom)], [*CIB2*: c.571C>T: p.(R191W) (Hom)]	Usher syndrome, type IJ
*ACTG1*	Fam20	c.94C>T	p.(P32S)	rs1598551290	AD	Hom		Baraitser–Winter syndrome 2
*SLC26A4*	Fam17	c.2089+1G>A	–	rs727503430	AR	Hom		Pendred syndrome
*SLC26A4*	Fam25	c.1225C>T	p.(R409C)	rs147952620	AR	Hom		Pendred syndrome
*Associated with syndromic HI only*								
*HARS2*	Fam16	c.346G>A	p.(A116T)	rs1312606802	AR	Het		Perrault syndrome 2
		c.659A>G	p.(Y220C)	rs746757469	AR	Het

**Table 3:** Novel non-syndromic and syndromic hearing impairment candidate genes.

Gene	F-ID	Phenotype	Nucleotide change	Protein change	Rs-number	Inh	GT	Expression in the mouse inner ear	Previous/animal studies
*INPP4B*	Fam2	NSHI	c.1848G>C	p.(Q616H)	rs147919355	AR	Het	Yes	Depletion of *INPP4B* suppresses callosal axon formation in the developing mice and may cause mild to severe cognitive impairment INPP4B regulates nerve conduction velocity Mouse models and rat models express the gene in the inner ear.
			c.1271T>C	p.(I424T)	rs747224392	AR	Het		
*CCDC141*	Fam26	NSHI	c.704A>G	p.(D235G)	rs1029313097	AR	Het	Yes	*CCDC141* variants have been implicated in Kallmann Syndrome, and though rare, HI is reported in some Kallmann Syndrome patients. Mouse models express the gene in the inner ear. Ccdc141*-*null mice showed impaired recognition memory and spatial reference memory.
			c.202G>A	p.(E68K)	rs540836199	AR	Het		
*MYO19*	Fam40	NSHI	c.949G>T	p.(A317S)	rs199866785	AR	Hom	Yes	Mouse models express the gene in the inner ear. The ortholog of the gene was associated with autistic disorder in different animals such as *Canis lupus familiaris, Pan paniscus, Sus scrofa*, and Chinchilla lanigera. In humans, Myosin 19 functions as an actin-based motor for mitochondrial movement in vertebrate cells.
	Case1	NSHI	c.1300G>A	p.(A434T)	rs375962449	AR	Het		
			c.2552C>T	p.(P851L)	rs749344013	AR	Het		
*DNAH11*	Fam44	NSHI	c.11232C>G	p.(I3744M)	rs201120788	AR	Het	Yes	*DNAH11* was implicated in primary ciliary dyskinesia and Kartagener syndrome which has HI as one of its symptoms.
			c.12969G>C	p.(Q4323H)	rs191802172	AR	Het		
	Case2		c.6118C>T	p.(R2040C)	rs199772877	AR	Het		
			c.846G>A:	p.(M282I)	rs375023124	AR	Het		
	Case3		c.7295G>A	p.(R2432Q)	rs769003090	AR	Het		
			c.6131G>A	p.(R2044Q)	rs372051486	AR	Het		
*SOX9*	Fam45	NSHI	c.432-3C>A	–	rs1033320617	AD	Het	Yes	*SOX9* is essential for cochlear development in mice. Mutant mice had a HI phenotype. Depletion of Sox9 resulted in defective vestibular structures, semi-circular canals, and utricle developments.
*PAX8*	Fam39	Waardenburg syndrome	c.968C>G	p.(P323R)	rs1573435665	AD	Het	Yes	(*Pax8*^−/−^ mice did not respond to sound when examined by the auditory brain stem response (ABR) test.
*POTEI*	Fam6	NSHI	c.1676G>C	p.(G559A)	rs1254207451	AR	Het	–	–
			c.409C>T	p.(R137X)	rs536831847	AR	Het		

Cases 1–3 are isolated cases (families with only one affected individual) that were examined as added participants from Ghana.

*Rs-number* reference number, *Inh* mode of inheritance, *hom* homozygote, *het* heterozygote, *GT* patient genotype.

### Bioinformatic and molecular analysis

#### Principal component analysis

**Fig. 2:**
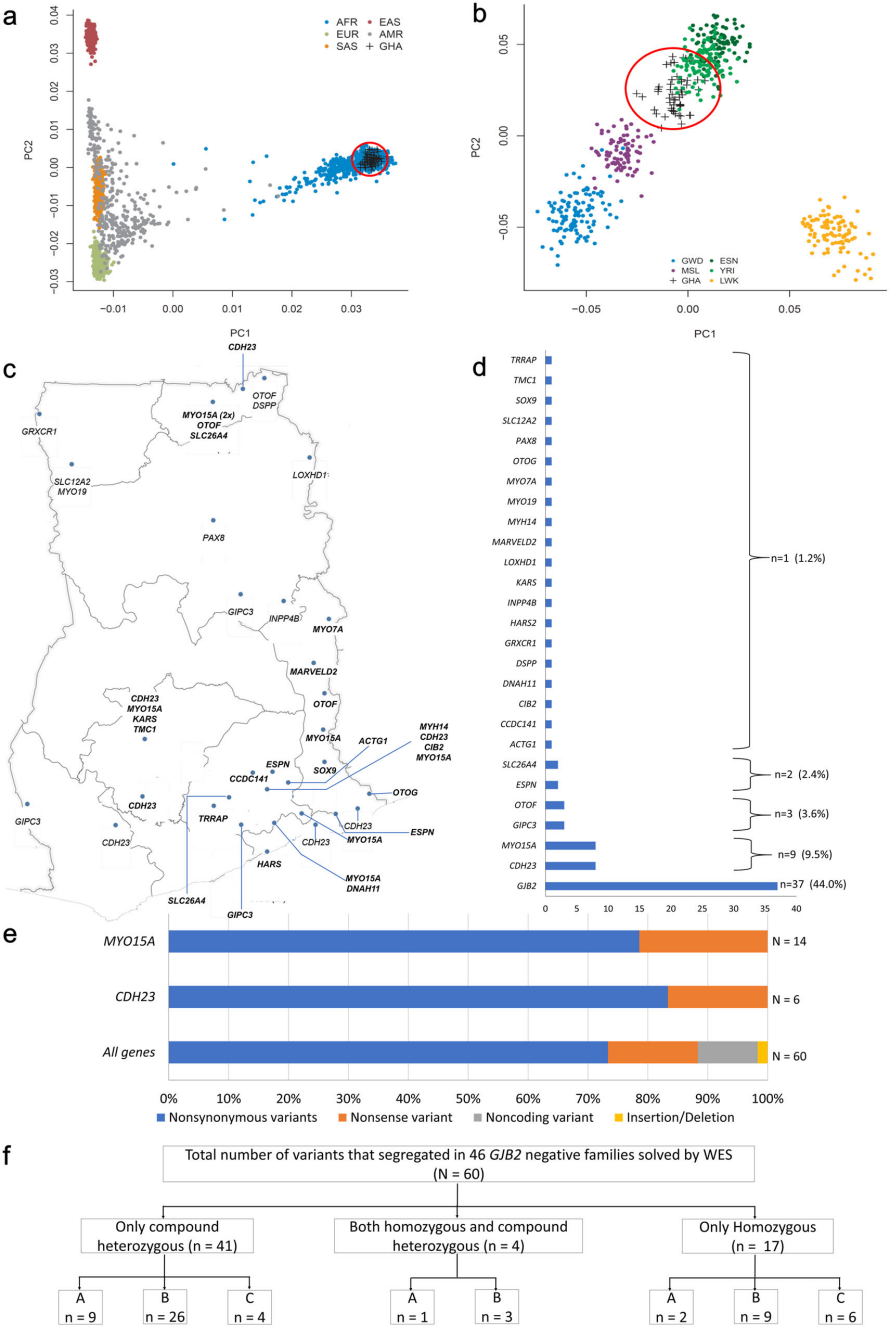
Principal component analysis and overview of gene variants found in Ghana. PCA plot of samples from Ghana projected against the **a** global populations and **b** African populations. GHA samples from Ghana (highlighted with red circles), AFR Africa, EUR European, SAS South Asian, EAS East Asian, AMR Admixed Americans, GWD Gambian in Western Division, MSL Mende in Sierra Leone, ESN Esan in Nigeria, YRI Yoruba in Ibadan, Nigeria, and LWK Luhya in Webuye, Kenya. **c** Geographical distribution and **d** frequency of the known HI and candidate genes in Ghanaian families. **e** Proportions of variant types in the two most prevalent (*MYO15A* and *CDH23*) and other known and novel candidate HI genes were identified. **f** A flow diagram showing variants absent from the databases “A”, present in the databases with new HI association “B” and known HI variants “C” identified in the families studied.

Principal components (PCs) constructed using genotype data obtained from families in this study were projected against data extracted from participants of continental ancestries in the 1000 Genomes reference panel (phase 3 version 5) showed that our samples clustered with other African populations, as expected (Fig. 2a). When projected only against African populations, our samples clustered between samples from Nigeria [YRI (Yoruba) and ESN (Esan)], and Sierra Leone [MSL (Mende)] as geographically expected, with closer proximity to the YRI population of Nigeria (Fig. 2b). Projecting all other family members against the coordinates of one member per family showed that members of the same family clustered close to each other (Supplementary Fig. 1h).

#### Known and candidate HI genes identified within the Ghanaian population

DNA samples from a total of 180 individuals from 51 *GJB2*-negative families underwent WES, including 104 hearing-impaired individuals and 76 unaffected family members. In these 51 families, variants in known human HI genes were found in 40/51 (78.4%) families, and highly likely candidate genes in 7/51 (13.7%). A total of 60 variants were found in 26 genes identified in the families studied (Fig. 2c, d and Tables 1, 2). The identified genes were randomly distributed across the territories of Ghana (Fig. 2c). Forty-four of the variants were missense variants and the remaining variants were insertion/deletion (1), nonsense (9), or non-coding variants (6) (Fig. 2e and Tables 1, 2). Of all the variants, 12 (20.0%) were not observed in dbSNP^16^, gnomAD^17^, Ensembl^18^, and TOPMed^19^ databases. Thirty-eight of the 60 variants (63.3%) were present in databases but were not previously reported to be associated with HI, including 24 of 48 (50.0%) variants in known HI genes. Only 10 (16.6%) of the observed variants were previously reported to be associated with HI (Fig. 2f).

Most families investigated (30/51) had unique variants (Tables 1–3), confirming the high level of allelic heterogeneity for HI genes within the Ghanaian population. Variants in *CDH23* (*n* = 8 families) and *MYO15A* (*n* = 8 families) accounted for the majority (31.4%) in this *GJB2*-negative group of families and represent 18.2% (16/88) of all families in the entire Ghanaian cohort initially recruited (Tables 1 and 2). *GIPC3* and *OTOF* variants were found in three families each, while variants in *ESPN* and *SLC26A4* were each found in two families, and variants in 20 other genes were only observed in one family (Tables 1, 2 and Fig. 2d). Thirty-one of the variants in known HI genes were classified as pathogenic (P) or likely pathogenic (LP) and 32/51 families were found to segregate at least one pathogenic and likely pathogenic (PLP) variant (Supplementary Table 1).

#### Non-syndromic hearing impairment gene variants

Fourteen known NSHI genes were identified in 25 of the 51 families (49.0%). These genes included *MYO15A* that was one of the most common genes observed (*n* = 8 families: Table 1). For the NSHI genes, 13 compound heterozygote variants were identified, and each variant was unique to a family except for *MYO15A* c.6011C>T: p.(P2004L) and c.8340G>A: p.(T2780T) that were found in two families (Table 1). The other 12 NSHI genes were *GIPC3*, *OTOF*, *ESPN*, *DSPP*, *GRXCR1*, *KARS*, *LOXHD1*, *MARVELD2*, *MYH14*, *OTOG*, *TMC1*, and *TRRAP* (Table 1).

#### Expansion of phenotypes for NSHI: detection of recessive inheritance for *DSPP*, and post-lingual expression of HI for MARVELD2

For *DSPP*, an ADNSHI gene, an AR mode of inheritance was detected in Fam37 (Table 1 and Supplementary Fig. 6).

For *MARVELD2*, we found a post-lingual expression of ARNSHI in Fam18, with variable age of onset with two family members with onset at 12 years of age and a third family member with onset at 8 years of age. All the affected family members could efficiently read lips, and since the condition developed post-language acquisition, they were able to engage in verbal communications (Table 1 and Supplementary Fig. 1).

#### Genes that cause both non-syndromic, and syndromic hearing impairment

Eight families segregated variants in *CDH23* (OMIM: 601067) (Table 2), which has been implicated in Usher syndrome type 1D^20^, ARNSHI^21^, and age-related HI^22^. All the variants (*n* = 6) identified in these families were classified as PLP based on the ACMG-AMP classification guidelines for HI (Supplementary Table 2). *CDH23*: c.4562A>G: p.(N1521S) and c.2746G>A: p.(D916N) were found in four and two families, respectively. The four families, which segregated the *CDH23*: c.4562A>G: p.(N1521S) variant, were ascertained in different geographical regions in Ghana. Three families were found to have compound heterozygote variants. In addition, Fam42 was found to have a *CIB2* (OMIM: 605564): c.556C>T: p.(R186W) variant segregating with the HI phenotype (Table 2). The above variant was classified as likely pathogenic with a combined annotation dependent depletion (CADD) score of 32, suggesting it is deleterious. The *CIB2* [c.556C>T: p.(R186W)] variant was previously found in an Usher syndrome type 1J family^23^, although it is currently questioned whether *CIB2* is involved in Usher syndrome etiology. Moreover, *MYO7A* (OMIM*:* 276903) is associated with Usher Syndrome type 1B (OMIN: 276900), ARNSHI (OMIM: 600060), and ADNSHI (OMIM: 601317), and PLP variants in this gene were found in Fam8 (Table 2). Two families (Fam17 and Fam25) segregate *SLC26A4* (OMIM: 605646) variants [c.2089+1G>A and c.1225C>T p.(R409C)]. *SLC26A4* has been associated with ARNSHI and Pendred syndrome [(OMIM: 274600) (Table 2)]. Fam20 segregates *ACTG1* (OMIM: 102560) variant c.94C>T: p.(P32S), *ACTG1* has been associated with ADNSHI, and AD Baraitser–Winter syndrome 2 BWS2 [(OMIM: 614583); (Table 2)]. BWS2 is mostly likely always due to de novo variants, contrary to the Fam20 pedigrees that feature ADNSHI with an affected mother, four affected and two unaffected children of both sexes (Supplementary Fig. 4). For the families that segregate variants in genes associated with both NSHI and syndromic HI, there were no clinical signs observed during sample collection that would indicate that affected family members have syndromic HI.

#### Hearing impairment gene associated with Perrault syndrome

*HARS2* compound heterozygote variants were identified as the likely cause of HI in Fam16 (Table 2). *HARS2* (OMIM: 600783) has been implicated in AR Perrault syndrome 2 (OMIM: 614926) that is characterized by HI in both males and females. Affected females also display primary amenorrhea, streak gonads, and infertility, while affected males show normal pubertal development and are fertile. Since the two HI family members are male, it is not possible to clinically diagnose this syndrome.

### Seven candidate HI genes

#### Six candidate genes identified in Ghanaian families with NSHI

**Fig. 3:**
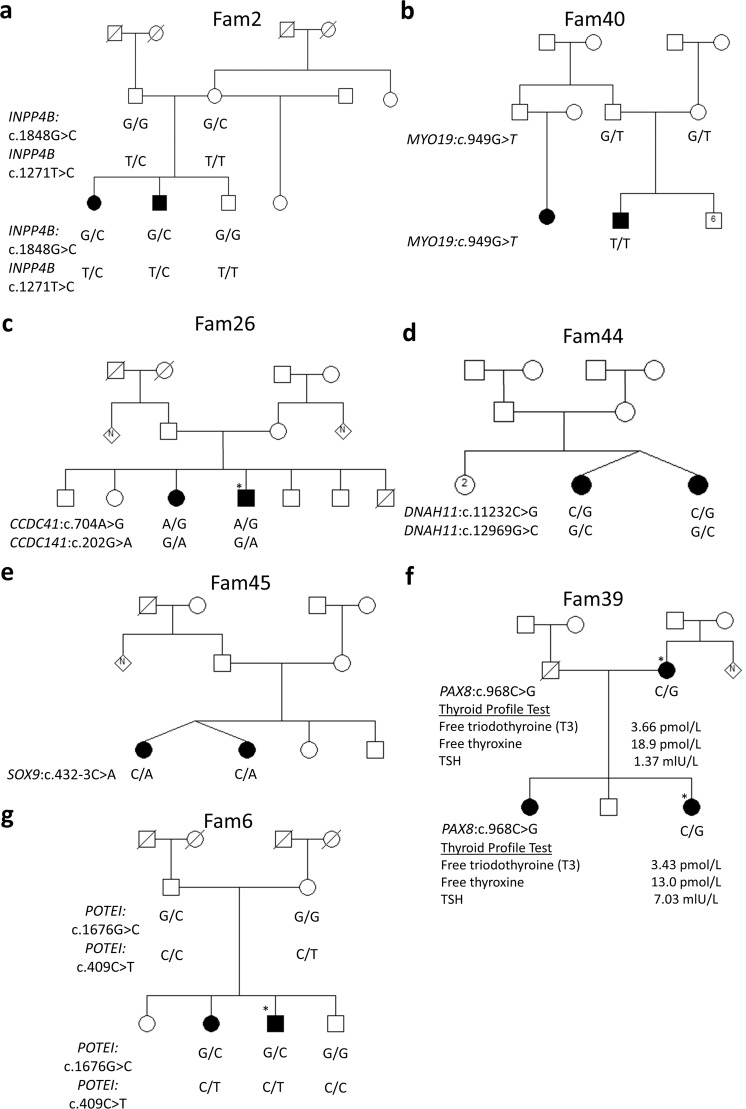
Pedigrees of families which segregate candidate genes. The segregation of candidate gene variants was shown in the respective families, **a** Fam2, **b** Fam40, **c** Fam26, **d** Fam44, **e** Fam45, **f** Fam39, and **g** Fam6. *Individuals whose audiogram was obtained.

Six genes (*INPP4B*, *CCDC141*, *MYO19*, *DNAH11*, *POTEI*, and *SOX9*) whose variants segregate with NSHI were found in six families (11.8%). Five of the six families had compound heterozygote variants which segregated with ARNSHI (Table 3, Fig. 3, and Supplementary Table 3).

Two variants were intronic: In family 40, a splicing variant in *MYO19* (c.2464-8T>C) segregated with HI but was found to be likely benign (Supplementary Table 4), but another biallelic missense variant in the same haplotype was classified as likely pathogenic (*MYO19:* c.949G>T). While a likely pathogenic splicing variant (*SOX9:* c.432-3C>A) was found in Fam45 segregated with an AD mode of inheritance (Supplementary Table 4). The gnomAD and TOPMed allele frequencies for these variants identified in candidate genes were either all zero or close to zero (Supplementary Table 3). CADD scores are also provided in Supplementary Table 3.

#### PAX8: a candidate gene for Waardenburg syndrome

A variant in a candidate gene [*PAX8*: c.968C>G: p.(P323R)] was found in one of the two families clinically presenting with Waardenburg syndrome (Table 3 and Fig. 3). The patients presented with the typical features of Waardenburg syndrome: HI, striking blue eyes, and premature gray hair phenotype (Supplementary Fig. 1f). Because, in addition to HI *Pax8* knockout mice model was reported to be athyroid^24^, we investigated thyroid hormones profile in the family and found it to be normal in the affected mother and daughter, except for thyroid-stimulating hormone that was high in the affected daughter, suggesting a trend to hypothyroidism (Fig. 3f). Although the variant was not in gnomAD it was present with a low frequency in TOPMed (minor allele frequency [MAF] = 1.13 × 10^−5^) and had a CADD score of 14.55. In addition, it has been reported that *Pax8*^−/−^ mice did not respond to sound when tested using auditory brain stem response (ABR). Moreover, abnormalities in the outer and middle ear structures were found in a high percentage of *Pax8*^−/−^ mice. Maturation of the inner ear also appeared delayed by about 1 week with respect to euthyroid controls^24^.

#### Other variants of unknown significance

Additional variants were found in some of the families, but these variants either did not segregate with the phenotype, or a second heterozygote variant was not found for families with ARHI. One of such families was Fam26 (Fig. 3c) in which the *LOXHD1* (OMIM:613072): c.4202C>T variant was found in the heterozygous state without a second variant. Fam39 (Fig. 3f) had only one affected family member positive for *HGF* (OMIM: 142409): c.776A>G; c.278G>C compound heterozygote variants. The same family member also had *FOXI1* (OMIM:601093): c.776G>A heterozygote variant. In addition, a heterozygote *COQ8A* (OMIM: 606980): c.692A>C variant was segregated in Fam39 but a second variant was not identified. In Fam40 (Fig. 3b), a heterozygote *MED12* (OMIM:300188): c.5348A>G variant was found. Compound heterozygote variants in *GRXCR1* (OMIM:613283): c.74G>A; c.645G>T were found in only one affected Fam45 member. Other heterozygote variants observed in Fam45 were *TECTA* (OMIM:602574): c.545A>G and *GREB1L* (OMIM: 617782): c.2498C>A.

#### Validation of candidate gene variants among isolated African HI cases

We investigated candidate genes in Ghanaian exomes data of non-familial/isolated HI cases (*n* = 153) and found two probands with compound heterozygous variants in *DNAH11* and in one proband with *MYO19* variants (Table 3 and Supplementary Fig. 7). Using Sanger sequencing, we validated the variants found in *DNAH11* and *MYO19* in the three probands (Supplementary Fig. 7).

#### Expression of HI candidate genes in the developing and adult mouse inner ear

**Fig. 4:**
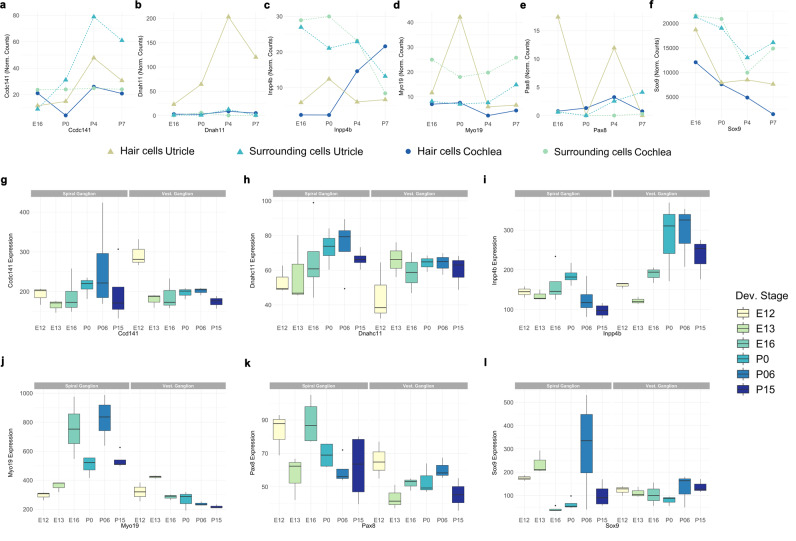
Expression of novel candidate genes in the cochlea and utricle during mouse development. **a**–**f** RNA sequencing data of hair cells and surrounding cells from the cochleae and utricles of mice at four developmental stages: E16, P0, P4, and P7 are presented for the mouse orthologs of six novel candidate genes^104^: *Ccdc141* (**a**), *Dnah11* (**b**), *Inpp4b* (**c**), *Myo19* (**d**), *Pax8* (**e**), *Sox9* (**f**). All genes show expression in the inner ear hair cells. Data are presented as normalized RNA-seq counts. **g**–**l** Expression of novel candidate genes in spiral and vestibular ganglion neurons of the inner ear during mouse development at six developmental stages: E12, E13, E16, P0, P06, and P15^105^. Expression is presented for *Ccdc141* (**g**), *Dnah11/ Dnahc11* (**h**), *Inpp4b* (**i**), *Myo19* (**j**), *Pax8* (**k**), and *Sox9* (**l**) with all genes showing expression in the spiral and vestibular ganglion of the inner ear during development. Expression microarray data is presented by the perfect match and mismatch probe differences (PM/MM). Data for (**a**–**l**) were obtained from SHIELD (Shared Harvard Inner-Ear Laboratory Database).

We investigated the expression of the six HI candidate genes in the inner ear via the interrogation of various expression atlases and found evidence of their expression. These genes all show expression in the inner ear hair cells, and in the spiral and vestibular ganglion during development [Fig. 4, *Ccdc141* (panels a and g), *Dnah11* (panels b and h), *Inpp4b* (panels c and i), *Myo19* (panels d and j), *Pax8* (panels e and k), *Sox9* (panels f and l)]. In addition, there was evidence of expression in various other cell types of the cochlear floor epithelium (Supplementary Figs. 8–16). *Myo19a* shows a wide-spread expression in the cochlear epithelium throughout E14, E16, and P1, and is predominantly expressed in the inner hair cells at P7 (Supplementary Figs. 8 and 13–15). *Sox9* shows a high and ubiquitous expression pattern across the entire cochlear epithelium, particularly during E14, E16, and P1. It displays a more restricted pattern during P7 with a predominant expression in the outer and inner pillar cells (Supplementary Figs. 9 and 13–15). C*cdc141* displays a wide-spread but low expression in the cochlear tissues throughout development, with high expression at E14 in the lesser epithelial ridge cells and the basilar membrane cells at P7 (Supplementary Figs. 10 and 13–15)*, Inpp4b* and *Dnah11* show a more restricted expression in the cochlear tissues throughout development, with *Inpp4b* expressed in the developing lateral great epithelial ridge cells at P1, the basilar membrane cells at P7; and *Dnah11* expressed in developing inner hair cells and mature inner hair cells at P1 and P7 respectively (Supplementary Figs. 11 and 12).

Moreover, five candidate genes i.e. *Ccdc141, Inpp4b, Myo19, Sox9, and Pax8* also showed expression in various craniofacial tissues during early mouse development. Specifically, there was the greatest expression of *Ccdc141, Inpp4b, Myo19*, and *Sox9* at E10.5, within the maxillary arch epidermal ectoderm for *Ccdc141*and *Inpp4b;* the lateral prominence neural epithelium for *Myo19*, and the central neural epithelium for *Sox9* (Supplementary Fig. 15). The greatest expression of *Pax8* occurs at E8.5 (Supplementary Figs. 15 and 16), within the caudal brain neural epithelium (Supplementary Fig. 15). *Dnah11* was not assessed in this latter data set and could not be interrogated.

In Fam6, compound heterozygous variants in *POTEI:* (c.1676G>C and c.409C>T) were found to segregate with ARNSHI. This gene does not have a mouse ortholog and expression data could not be investigated in the available data sets.

#### Protein structure analysis

**Fig. 5:**
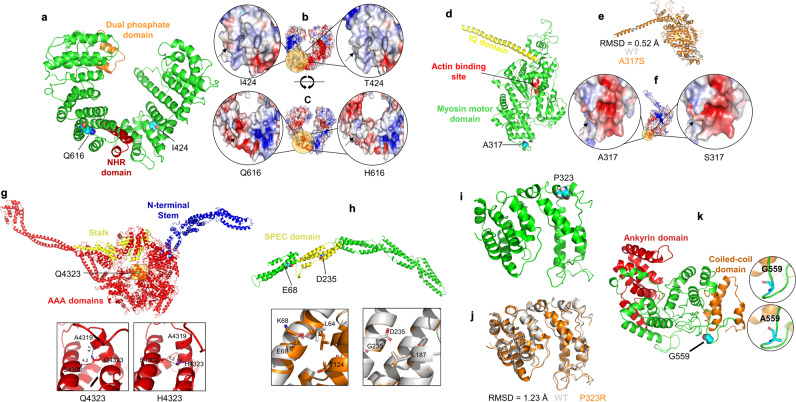
Structural modeling missense variants in novel hearing impairment candidate genes. **a** Homology model of INPP4B. The mutated residues (I424T and Q616H) are represented with a sphere model. The NHR and dual phosphate domains are indicated with red and orange colors, respectively. **b**, **c** The electrostatic potential surface of wild-type and mutant protein is highlighted in the circle zoom-up view showing different patches (indicated by the arrow) around the mutated residue. **d** Three-dimensional structure of MYO19 showing the mutated residue, A317, in a sphere model. The actin-binding pocket, myosin motor, and IQ domain are represented by green, red, and yellow colors, respectively. **e** The superposed structure of the WT (gray) and mutant (orange) structure of MYO19 shows the root-mean-square deviation. **f** The electrostatic potential surface of wild-type and mutant protein is highlighted in a circle zoom-up view showing different patches (indicated by the arrow) around the mutated residue. **g** Three-dimensional structure of DNAH11 showing the N-terminal stem (blue), stalk (yellow), and the mutated residue Q4323 (sphere model) of the AAA domain (red). The difference in the interaction network due to the mutation is highlighted in the bottom panel. The residue is represented with a stick model and the distance between the residues is labeled in angstrom. **h** Predicted structure of CCDC141 showing the mutated residues in a sphere model. The bottom row represents the zoom-up view of WT (gray) and mutant type structures displaying the different orientations of residues (stick model). **i** Three-dimensional structure of PAX8. **j** The superposed structure of WT (gray) and mutant (orange) structure showing the difference in overall conformation due to the P323R mutation. **k** Predicted structure of *POTEI* showing the wild-type and mutated residues in a stick model in the right panel.

Homology modeling techniques were utilized to model the wild-type (WT) and the mutant missense variant(s) of three-dimensional structures of INPP4B, MYO19, DNAH11, CCDC141, and PAX8 proteins via the MODELLER program^25^. In the case of INPP4B, the two mutated residues, I424 and Q616, reside at the helix and loop region, respectively (Fig. 5a). Both residues are located on the surface of the protein and do not interact directly with nearby residues. The substitution of nonpolar hydrophobic amino acid isoleucine into polar residue threonine at position 424 displays different surface patches of electrostatic potential (Fig. 5b). In the case of Q616H, the replacement of neutral polar charged glutamine into basic polar charged histidine causes a shift in electrostatic potential and surface area, as indicated by the arrow (Fig. 5c).

For *MYO19*, residue A317 is located at the surface of the protein in the myosin motor domain (Fig. 5d). The RMSD of WT (A317) and mutant (S317) MYO19 show a value of 0.52 Å, suggesting a slight change in overall conformation (Fig. 5e). The electrostatic potential of mutant protein compared with WT displays a change in surface charge distribution as shown in Fig. 5f.

The mutated residue Q4323 in DNAH11 is located in the helix region of the AAA domain (Fig. 5g). The Q4323 residue is involved in an interaction with nearby residues, A4319, and S4302. The substitution of WT glutamine with a histidine residue changes the interaction network of nearby residues. We were unable to reliably model the I3744M variant for DNAH11.

For CCDC141, E68K and D235G both alter the nature of the side chain (Fig. 5h). The close views of WT and mutant CCDC141 protein display the change in the secondary structure and side chain of the nearby residues.

The superimposed structure of the WT and mutant structure of PAX8 structure displays a 0.52 Å RMSD value suggesting the effect of the mutation on the overall conformation (Fig. 5i, j).

The POTEI p.(G559A) missense variant was modeled (Fig. 5k) and the predicted structure shows a minimal difference between the WT and mutated residues. The p.(R137X) variant in *POTEI* was not modeled as that one is predicted to lead to nonsense-mediated decay and there is likely no protein expression.

### Unsolved families

In four families (7.8%), a possible gene for HI was not identified. Although rare variants in HI genes were identified in the exome sequence data for these families, they were unlikely to be the cause of HI (Supplementary Table 5 and Supplementary Fig. 17). An example is one family presenting with Waardenburg syndrome (Fam51) that had variants in three HI genes, but these variants did not segregate with the phenotype (Supplementary Table 5). Assuming an AD inheritance model (with a possible affected father), we did not find any variants in a known HI or Waardenburg gene. There are multiple possible candidate variants that will need confirmation in the future (Supplementary Table 5).

Fam4 segregates a previously reported *BCORL1* (OMIM:300688) variant c.4079_ 4081del: p.(D1360_ L1361delinsV) with HI. *BCORL1* causes Shukla–Vernon syndrome (OMIM: 301029), an X-linked recessive neurodevelopmental disorder characterized by global developmental delay, variably impaired intellectual development, and behavioral abnormalities, including autism spectrum disorder and ADHD^26^. This variant is unlikely to be responsible for HI because it has not been reported as part of this syndrome before. Moreover, Fam4 features NSHI. In addition, there is no inner ear expression data available for this gene. Fam4 segregates a heterozygous *MYO15A* variant that could have explained the HI, but a second variant could not be identified (Supplementary Fig. 17).

Fam48 had a heterozygous *GJB2* p.(R143W) founder variant in one of the two affected family members, but a second *GJB2* variant was not identified (Supplementary Table 5).

In Fam52, we identified compound heterozygous variants segregating with HI in *DACT2* (c.796-12C>T and c.1021T>G). DACT2 is an important mediator involved in the regulation of intracellular signaling pathways during development^27^. DACT2 is expressed in the outer pillar cells of the inner ear^28,29^, but its role in HI remains to be fully characterized. Moreover, the CADD score was low for one of the variants found in *DACT2*, and the other variant is located in the splicing region with no predicted impact on splicing. Also, for Fam52, compound heterozygous variants were found to segregate with HI in *NCL* [c.2065G>A: p.(G689S), and c.188A>G: p.(K63R)]. *Ncl* is widely expressed in the mouse and zebrafish ear. However, there is not enough evidence to support it as a good candidate and one of the variants was also found in the heterozygous state in three members of another family in this study, suggesting its frequency may be high in this population.

Last, no relevant copy number variations (CNVs) encompassing HI genes were identified in any of these families.

## Discussion

In this study, we investigated the largest exome sequence data set for sub-Saharan African families segregating HI. This study will inform and enhance genetic medicine practice locally in Ghana, and African populations in the diaspora, with putative West African/Ghanaian ancestry. The principal component analysis (PCA) plot shows the similarity between the data obtained from the studied families and those obtained for the Nigerian population (Fig. 2a, b). Our previous investigations identified *GJB2*: p.R143W variant as the most frequent cause of familial NSHI in Ghana^3^. Putting together, the *GJB2* positive rate from our previous studies^3,13^ and some additional families, we re-evaluated the contribution of *GJB2* to 42.0% (37/88) among all families investigated (Fig. 1a). However, the majority of *GJB2* positive cases originated from the same region (Eastern Region) where the village with a high prevalence of deafness (Adamorobe) is located^11,13^. A frequency rate of 34.1% was estimated without the inclusion of cases from Adamorobe village.

The present data identified variants in 20 known HI genes of which *CDH23* (8/88; 9.1%) and *MYO15A* (8/88; 9.1%) were observed to be the cause of NSHI in multiple families, supporting future prioritization of these two genes in clinical practice. Moreover, the overall combined frequency of 18.2 % (16/88) for *CDH23* and *MYO15A* amongst hearing-impaired families in Ghana is likely underestimated due to an oversampling of *GJB2* positive families, with the frequency amongst *GJB2*-negative families at 31.4% (16/51). Indeed, we probably oversampled families from the Eastern region of Ghana where the *GJB2-*p.(R143W) founder variant was first described and is more prevalent^3,11,12^, and consequently where most schools for the deaf are established (Fig. 1b).

Similar to a previous study in Cameroon using a targeted panel sequencing^30^, within the Ghana population there is a high degree of locus and allelic heterogeneity. Five out of the seven families (71.4%) solved in the Cameroonian study had compound heterozygote variants in the identified gene, likely due to the absence of consanguinity. In the current report, 22/47 solved (46.8%) *GJB2*-negative families display compound heterozygosity. This will pose a great challenge in the development of a single affordable diagnostic tool that can be widely used in clinical practice, specifically for compound heterozygotes, which are harder to detect than homozygous variants and also parents need to be investigated to ensure when two variants within a gene are on different haplotypes. Targeted sequencing panels are useful in diagnosing genetic HI, however, only known genes can be investigated. In addition, there is a need for constant upgrade and revalidation of the targeted sequencing panels whenever new HI genes are discovered^31,32^. The above limitations, and the evidence illustrated by the present study advocate for the use of clinical WES and whole-genome sequencing^31,33–36^. It is also worth noting that additional variants in known HI genes as found in this study (Tables 1 and 2) can complicate interpretation. In addition, the handling of incidental findings, in so-called actionable genes, and policies toward them is still to be implemented in African settings^37,38^.

*CDH23* has been implicated in ARNSHI and Usher syndrome type 1D (Table 2)^20^, and was reported as an important contributor to HI in several global populations^39,40^ with high frequencies in Eastern Asian countries such as Japan^41^ and Korea^42^. Two of the identified *CDH23* variants were previously associated with HI, c.5237G>A: p.(R1746Q) in Spain^43^ and Ireland^44^ and c.2206C>T: p.(R736X) in French Canadians from Quebec^45^.

*MYO15A* has been ranked as the third to the fourth most common cause of severe-to-profound ARNSHI, worldwide^46,47^. The global frequency of HI-associated *MYO15A* variants was calculated at 6.2% by Farjami et al. underscoring the importance of this gene in the development of HI^48^. The frequency observed in Ghana is higher than what was previously reported for some African countries such as Nigeria (2.0%), South Africa (2.0%), Tunisia (2.3%)^49^, and Morocco (3.0%)^50^ using a custom capture panel (MiamiOtoGenes) and WES. On the other hand, one study of 61 Egyptian families reported a frequency of 15.3%^51^. Recent WES data from a Cameroonian study identified variants in *MYO15A* in 22.2% of hearing-impaired cases of putative genetic origin; with three variants identified in multiplex families and one variant detected in a simplex family. These data suggest that MYO*15A* may be the most common gene underlying HI in Cameroon^52^. A review on *MYO15A* has shown that the majority of studies reported novel variants^48^, which is consistent with our study. To the best of our knowledge, we identified four novel *MYO15A* variants [c.4778A>G:p.(E1593G), c.6551G>C:p.(C2184S), c.9947A>G:p.(Q3316R), and c.6518C>T:p.(S2173F)]. The large number of exons in *MYO15A* coupled with the fact that more than 2000 missense variants have been identified in this gene^46^, although not all involved in HI, makes routine screening of *MYO15A* variants in patients difficult.

An important novelty of this study is the identification of seven new HI candidate genes, six of which are associated with NSHI, and the AD variant, *PAX8*: c. 968C>G: p.(P323R), found in a family with Waardenburg syndrome. The involvement of these candidate genes in HI is also supported by clinical and segregation analyses (Table 3 and Fig. 3). Interestingly, variants in two of the candidate genes were also observed in unrelated HI patients from Ghana (i.e., *DNAH11, MYO19*). In the latest genome-wide association data browser from the FinnGen research project (Release 5; 218,792 individuals; https://r5.finngen.fi/), which aims to study genetic variation associations with various traits in the isolated population of Finland, we found variants in or near these seven novel candidate genes that are possibly associated to HI as well (+/− 100 kb). This includes variants in or near *CCDC141* (rs144697379; *p* = 2.6 × 10^−5^; intronic); *DNAH11* (rs2965393; *p* = 4.5 × 10^−4^; intronic); *PAX8* (rs115708270; *p* = 3.6 × 10^−4^; upstream); and *SOX9* (rs16977126; *p* = 5.7 × 10^−5^; downstream) for sensorineural HI, and in *MYO19* (rs143245472; *p* = 6.6 × 10^−6^; upstream); *INPP4B* [rs184880581; *p* = 2.6 × 10^−5^; intronic) and *POTEI* (rs1337496945; *p* = 5.6 × 10^−4^; downstream) for sudden idiopathic HI. Although these results support our findings that these genes are involved in HI, without access to the data it is impossible to correct for multiple testing which may render the finding insignificant. Moreover, unlike congenital HI described in Ghanaian patients, most of these participants are adults suggesting these genes could be also associated with late-onset HI.

For six candidate genes, publicly available RNA sequence and microarray data sets were used for in silico investigation of gene expression in the developing and adult mouse inner ear (Fig. 4 and Supplementary Figs. 8–13, *Ccdc141*, *Dnah11*, *Inpp4b*, *Myo19*, *Pax8*, and *Sox9*) and also for protein modeling studies (Fig. 5).

Paired box gene 8, *PAX8* (OMIM:167415), is located on chromosome 2q14.1 and encodes a transcription factor^53^. Cloning and expression experiments have shown that *PAX8* is mainly expressed in the thyroid gland and can cause hypothyroidism^54^. In addition, in *Pax8* mutant mice, major abnormalities of the outer and middle ear structures were found and an overall delay in the maturation of the auditory system of *Pax8* knockout mice compared to the WT mice^24^.

Previous studies have shown that SOX9 is part of a family of transcription factors that are involved in the regulation of inner ear development. In mice, Sox9 is required for invagination of the otic placode^55^ and the loss of Sox9 severely compromises expansion, differentiation, and remodeling of the otic fibrocyte compartment^56^. Sox9‐depletion also results in major defects in the development of vestibular structures, semi-circular canals, and utricle in *Xenopus* and in failure of otic placode invagination in the mouse^55,57^. In humans, previous molecular studies showed that hair cell differentiation in humans was consistently present from 12 weeks, coinciding with downregulation of SOX9^58^. Heterozygous variants in *SOX9* cause campomelic dysplasia (OMIM: 114290) that can include HI as one of its clinical expressions. However, campomelic dysplasia is often lethal in the first year of age, due to respiratory insufficiency related to small chest size life and tracheobronchial hypoplasia. Moreover, the Ghanaian family with monoallelic variant in *SOX9* only presented with HI and there was no evidence of shortness and bowing of long tubular bones, or sex reversal. Nevertheless, a careful follow-up with an evaluation of evolving discrete phenotypes of campomelic dysplasia could be considered in this family.

There is a relatively large number of HI genes that encode myosin superfamily members (*MYO3A, MYO6, MYO7A, MYO15A, MYH14*, and *MYH9*)^59^; therefore, it might not be surprising that *MYO19* is a HI gene. Human myosin 19 functions as an actin-based motor for mitochondrial movement in vertebrate cells and promotes their localization to stress-induced filopodia^60–62^. In mice, Myo19 is a high-duty ratio molecular motor moving to the plus-end of the actin filament^63^.

A previous study in mice has indicated that C*cdc141* is required for radial migration and myosin II-mediated centrosome positioning during neuronal development^64^. Loss-of-function analysis in primitive neural stem cells derived from mouse embryonic stem cells demonstrated that Ccdc141 plays an important function in the regulation of early neural commitment^65^. *Ccdc141* mouse mutants have normal ABR thresholds, but there are other possible explanations for this, such as incomplete knockout of gene function. Ccdc141*-*deficient mice exhibited impaired recognition memory and spatial reference memory^66^. In human, biallelic variants in *CCDC141* was associated with pituitary stalk interruption syndrome (a rare disorder characterized by an absent or ectopic posterior pituitary, interrupted pituitary stalk, and anterior pituitary hypoplasia)^67^; and in normonosmic or anosmic hypogonadotropic hypogonadism, due to neuronal migration disorders the results in a defect in the development of the GnRH and the olfactory system^68–70^.

Dynein heavy chain 11 (DNAH11), an essential component of ciliary structure and function and left-right asymmetry in mice^71–73^. Biallelic variants in the *DNAH11* gene are associated with primary ciliary dyskinesia in multiple populations^74–77^, and in heterotaxy syndrome, and laterality defect^78–80^. The present study adds NSHI to the spectrum of pathologies associated with variants in *DNAH11*, likely due to the alteration of primary cilia in hearing function found in numerous ciliopathies^81^.

*INPP4B* overexpression is associated with human achalasia, which is a rare motility disorder characterized by myenteric neuron and interstitial cells of Cajal abnormalities^82^. Depleting *INPP4B* by in utero electroporation in mice suppressed medially directed callosal axon formation and significantly attenuated the formation of pyramidal neurons and axon polarization in cortical neurons during cortical development^83^. However, the mechanisms of *INPP4B* dysregulation in hearing function will require further investigation.

In the future, it is desirable to perform in vitro functional analysis, particularly for the 17 missense and the one splice variants found in the novel candidate genes (Table 3), and if possible, to develop and investigate animal models when applicable, to complement the data provided in this study: families’ variants segregations (Fig. 3 and Supplementary Fig. 7), in silico analysis (Fig. 5 and Supplementary Table 4), and gene expressions profiles in the inner ear (Fig. 4 and Supplementary Figs. 8–16). It is also worthwhile to refine the phenotyping for the existing mutant hearing-impaired mice, i.e., *Pax8*, *Sox9*, and *Ccdc141*, with regard to the new clinical evidence described in this study that strongly supports their relevance to the genetics of human hearing.

In addition to the seven new candidate genes identified, a phenotypic expansion was seen for *MARVELD2*, and a family was found with a different inheritance model of *DSPP*. A high proportion of HI-associated variants that were not previously described was seen as well. Other strengths of this study are the number and the size of families and the ability to recruit a substantial portion of the family members. Exome sequence data generated for multiple family members facilitated segregation analysis and variant identification. There was no significant difference in the size of the solved versus unsolved families. The data set contributes to refining HI gene curation since most of the identified variants have not previously been associated with HI. The data will also contribute to available exomes from African populations, facilitate future exploration of pathogenic variants in actionable genes and refine their relevance and importance in African populations.

Using WES, this study of families from Ghana obtained a high-solve rate of genetic causes among *GJB2-*negative families segregating HI. WES should be considered for routine investigations in clinical settings. Six new genes were associated with NSHI: *INPP4B*, *CCDC141*, *MYO19*, *DNAH11*, *SOX9*, and *POTEI;* and one new gene, *PAX8*, was associated with Waardenburg syndrome. The study reveals and emphasizes the high level of allelic heterogeneity for HI genes. Most identified variants, 48/60 (80.0), had not previously been reported to be associated with HI, and to the best of our knowledge, 12 (20%) of the variants are also novel. Most of the *GJB2*-negative families that had variants in a known HI gene (29/48) had a unique variant segregating with HI, suggesting that future clinical diagnostic approaches should use next-generation sequencing, and ideally clinical WES for *GJB2*-negative families. This study will contribute to the global knowledge on the genetics of HI in understudied African populations and has provided the opportunity for novel HI genes discovery.

## Methods

### Ethical approval

We observed and adhered strictly to the guiding principles of the Declaration of Helsinki. Ethical approvals were obtained from Noguchi Memorial Institute for Medical Research Institutional Review Board (NMIMR-IRB CPN 006/16-17) and College of Basic and Applied Sciences, Ethics Committee for Basic and Applied Sciences (ECBAS 053/19-20) the University of Cape Town, Faculty of Health Sciences’ Human Research Ethics Committee (HREC 104/2018), and the Institutional Review Boards of Columbia University (IRB-AAAS2343). The study was clearly explained to each participant in the language in which they are fluent and informed consent was signed prior to participation. Parents/guardians provided signed consent for their children who were ≤18 years of age. In addition, assent was obtained for children older than 7 years of age. Where applicable, informed consent was obtained from the study participants for publishing their images.

### Participants’ recruitment

Patient recruitment procedures were previously described^3^. The probands of Ghanaian families segregating HI were identified through nine schools for the deaf across the country (Fig. 1), and additional family members were recruited thereafter. Families with HI were also identified and recruited through our community engagement activities^13^ (Fig. 1). Medical records of all our participants were reviewed by a medical geneticist, and an ear, nose, and throat specialist. Detailed personal and family histories were obtained through a rigorous clinical interview. A structured questionnaire was used to interview each participant to rule out potential environmental causes of HI. The studied families had at least two members affected with HI that followed a Mendelian mode of inheritance. For those participants that were ascertained from schools for the deaf, before being admitted to the school they had undergone a systemic general and otological examination, that included pure tone audiometry.

A total of 5 ml of peripheral blood was obtained from each participant and genomic DNA (gDNA) was extracted from the samples using the QIAamp DNA Blood Maxi Kit^®^ (Qiagen, USA).

### Exclusion of *GJB2* and the del(*GJB6*-D13S1830) deletion

All hearing-impaired participants were first screened using targeted sequencing as described previously^3^, and shown to be negative for PLP *GJB2* variants including the del(*GJB6*-D13S1830) deletion. Allele-specific primers were used to amplify the coding region of *GJB2* and *GJB6*. By the use of BigDye™ Terminator v3.1 Cycle Sequencing Kit (ThermoFisher Scientific), the amplicons were Sanger sequenced and resolved using ABI 3130XL Genetic Analyzer (Applied Biosystems, Foster City, CA, USA). A total of 88 families were ascertained altogether and 37/88 were found with *GJB2* variants and excluded from the set of 51 families to be studied by WES (Fig. 1a).

### Whole-exome sequencing and data analyses

gDNA samples from 51 families with at least two affected hearing-impaired family members (Fig. 1) underwent WES. The DNA concentration and quality checks were conducted using the QuantiFluor dsDNA System on a Quantus Fluorometer (Promega, Madison, WI). The exome library for 129 samples (batch one) was prepared using the Nextera Rapid Capture Exome kit (Illumina, San Diego, CA), 50 ng of gDNA was fragmented using the Nextera transposomes and the resultant libraries were hybridized with a 37 Mb probe pool to enrich exome sequences. Libraries were sequenced on an Illumina HiSeq 2500 sequencer (Illumina, San Diego, CA) with the use of the pair-end 100 bp run format. The exome library preparation of the rest of the samples *(n* = 51, batch two) was performed using SureSelect V4 + UTR 71 Mb All Exon Capture Kit (Agilent Technologies, Inc., Santa Clara, CA, USA), ~3–5 µg of the DNA was fragmented with ultrasound using a Covaris^®^ instrument (Covaris, Inc., Woburn, MA, USA). The libraries were sequenced on the Illumina HiSeq 2000 (Illumina, San Diego, CA) and produced paired-end reads of 100 bp. The exome sequence data mapping and variant calling were performed using the Illumina BaseSpace app suite. The Illumina DRAGEN Germline Pipeline v3.2.8 was used to align the sequence reads to the human reference genome (hg19) and variants were jointly called using the Genome Analysis Toolkit (GATKv4.1.7) software package^84^. Variant quality was assessed using variant quality score recalibration (VQSR) using the ApplyVQSR function of GATK.

### Annotation and filtering strategy

An in-house pipeline that uses ANNOVAR^85^, dbNSFP^86^, and dbscSNV was used to annotate and filter single-nucleotide and insertion/deletion (indels) variants. Filtering was performed using Genome Aggregation Database (gnomAD)^17^ population-specific MAF of <0.005 [for AR and X-linked (XL)] and <0.0005 for AD with variants meeting these criteria being further ranked based on the bioinformatics prediction scores from SIFT^87^; polymorphism phenotyping v2 (PolyPhen-2)^88^ MutationTaster^89^; CADD^90^; deleterious annotation of genetic variants using neural networks (DANN)^91^; and Genomic Evolutionary Rate Profiling (GERP++)^92^. Information from the Hereditary Hearing Loss Homepage (HHL)^6^, Online Mendelian Inheritance in Man (OMIM)^93^, Human Phenotype Ontology (HPO)^94^, and ClinVar^95^ databases and deafness animal models were also used to prioritize identified variants. The MAFs of variants were further evaluated using the TOPMed Bravo database^96^. In families for which candidate variant(s) were not identified exome sequence data were also analyzed for CNV using CoNIFER^97^. These variants were assessed for their clinical significance based on the ACMG-AMP classification^98^, considering CADD scores^90^, and their allele frequencies in gnomAD and TOPMed databases (Supplementary Tables 1 and 2).

### Principal component analysis (PCA)

Joint calling was performed on samples in each batch separately and two VCF files were generated that included only variants with read depth >8, genotype quality >20%, and that passed VQSR filters. Biallelic single-nucleotide variants (SNVs) for both batches (176,820 for batch one and 326,250 for batch two) were then extracted into separate VCF files. Further quality control to remove variants that were present in only one batch was performed by applying a genotyping call rate of 95%. The phased batch one and two data sets were then merged using bcftools and contained 1,141,838 biallelic SNVs of the phased 1KGP3 data, that were polymorphic in the Mende population from Sierra Leone (MSL). The MSL population has been previously reported to harbor a large proportion of basal West African ancestry^99^. Further quality control includes the removal of variant sites missing more than 5% of their genotypes and samples missing more than 10% of their data, as well as the removal of variants that failed the Hardy–Weinberg equilibrium test at a threshold of 1 × 10^−6^, and SNVs with MAF < 0.05 was applied to the merged data set using PLINK2^100^ leaving a total of 18,880 SNPs. Linkage disequilibrium-pruning including only SNPs with *r*^2^ < 0.2 within a 50 bp region (window size = 10 bp) left 14,987 high-quality SNVs for inclusion in the PCA, for which five PCs were computed using PLINK2.

### Expression of candidate genes in the developing and adult mouse inner ear

Various publicly available RNA sequencing and microarray data sets were used for an in silico investigation of the expression of *CCDC141, DNAH11, INPP4B, MYO19, PAX8*, and *SOX9* in the developing and adult mouse inner ear. To study the expression during mouse craniofacial development, series GSE55966 from the Gene Expression Omnibus database was evaluated^101,102^. This data set includes RNA sequence data of CD1 mouse embryos at three stages: E8.5, E9.5, and E10.5^102^. Craniofacial gene expression data were presented as a set of FPKM (fragments per kilobase million) values for 13 different tissue/stage pairs^102^ that were converted to TPM (transcripts per kilobase million) values for our analysis.

We also studied expression levels during mouse inner ear development in previously generated data sets present in the Shared Harvard Inner-Ear Laboratory Database (SHIELD)^103^. The first data set detailed expression over four developmental stages: E16, P0, P4, and P7^104^. Data were obtained from the cochlea and utricles of mice that expressed EGFP under the Pou4f3 promoter^104^. Fluorescence-activated cell sorting was used to separate cells into hair cells (GFP+) and surrounding cells (GFP–) prior to RNA extraction^104^. To supplement these data, a second data set was downloaded, which contained expression data for six developmental stages: E12, E13, E16, P0, P6, and P15^105^. The expression data were produced by Affymetrix Mouse 420 v.2 GeneChips and subset into expression in spiral ganglion neurons and vestibular ganglion neurons^105^.

Lastly, the gene Expression Analysis Resource (gEAR) suite was used to visualize expression in cells of the cochlear epithelium during mouse development. gEAR includes single-cell RNA sequence data obtained from CD1 mouse embryos at four developmental stages: E14, E16, P1, and P7^106^. *CCDC141*, *DNAH11*, *INPP4B*, *MYO19*, and *SOX9* expression was also grouped based on cell groups in four overarching classes: developing supporting cells, developing prosensory cells, developing sensory cells, and developing greater epithelial ridge cells of which each were further divided into subclasses^106^.

### Sanger sequencing validation of *MYO19* and *DNAH11* variants

Allele-specific primers (Supplementary Table 6) were designed using NCBI primer BLAST^®^ and optimized for polymerase chain reaction (PCR) and sequencing of the regions of *MYO19* and *DNAH11*. The initial denaturation temperature was 95 °C for 3 min and 30 s for subsequent denaturation. An annealing temperature was 60 °C for 30 s and an extension temperature of 72 °C for 1 min for 35 cycles was employed. The PCR amplicons were sanger sequencing using BigDye™ Terminator v3.1 Cycle Sequencing Kit (ThermoFisher Scientific). The Sanger sequences were resolved using ABI 3130XL Genetic Analyzer (Applied Biosystems, Foster City, CA, USA).

### Homology modeling

The human INPP4B (ID: O15327), MYO19 (Q96H55), DNAH11 (ID: Q96DT5), CCDC141 (ID: Q6ZP82), and PAX8 (ID: Q06710) protein sequences were obtained from the UniProt database for homology modeling^107^. To find appropriate structural templates for these proteins, PSI-BLAST was run against the Protein Data Bank (PDB). The crystal structure of PDB ID: 2Q5D^108^, PDB ID: 5IOI^109^, PDB ID: 3VKG^110^, PDB ID: 3EDV^111^, and PDB ID 4K6J^112^ were used as a template for the construction of INPP4B (residue 215-877), MYO19 (residue 30-804), DNAH11 (residue 1372-4515), CCDC141 (residue 36-562), and PAX8 (residue 120-398) protein models. Homology modeling was carried out for WT and mutant proteins using MODELLER^113^ based on the sequence alignment generated between the template and target sequences. The Adaptive Poisson-Boltzmann Solver Electrostatics plugin of PyMOL was used for protein surface electrostatic potentials. PyMOL^114^ version 2.0.3 was used for visualization and the processing of figures.

### Reporting summary

Further information on research design is available in the Nature Research Reporting Summary linked to this article.

## Supplementary information

Peer Review File

Supplementary Information

Description of Additional Supplementary Files

Supplementary Data 1

Supplementary Data 2

Reporting Summary

## Data Availability

The Sanger sequences generated were submitted to GenBank with the following accession codes: OM965691, OM965692, OM965693, OM965694, OM965695, and OM965696. SNV data have been submitted to dbSNP (https://www.ncbi.nlm.nih.gov/snp/). Source data for the graphs can be found in Supplementary Data 1 and Supplementary Data 2. All other relevant data supporting the key findings of this study are available within the article and its Supplementary Information files. Due to lack of ethical approval, individual-level whole-exome sequence data cannot be made publicly available; however, they can be obtained from the corresponding authors [A.W. and S.M.L.] upon reasonable request.
