# Supplementary material for: Exome sequencing of families from Ghana reveals known and candidate hearing impairment genes
Source: Commun Biol. 2022 Apr 19;5:369. doi: 10.1038/s42003-022-03326-8 (PMC9019055; doi:10.1038/s42003-022-03326-8)
Supplement: Supplementary file 2 — Supplementary Information [file 42003_2022_3326_MOESM2_ESM.pdf]

**Supplementary figures and tables**

***Supplementary figures***

Supplementary Figure 1 ……………………………………. page 2
Supplementary Figure 2 ……………………………………. page 4
Supplementary Figure 3 ……………………………………. page 5
Supplementary Figure 4 ……………………………………. page 6
Supplementary Figure 5 ……………………………………. page 7
Supplementary Figure 6 ……………………………………. page 8
Supplementary Figure 7…………………………………….. page 9
Supplementary Figure 8 ……………………………………. page 10
Supplementary Figure 9…………………………………….. page 11
Supplementary Figure 10……………………………………. page 12
Supplementary Figure 11……………………………………. page 13
Supplementary Figure 12……………………………………. page 14
Supplementary Figure 13……………………………………. page 15
Supplementary Figure 14……………………………………. page 16
Supplementary Figure 15……………………………………. page 18
Supplementary Figure 16……………………………………. page 19
Supplementary Figure 17……………………………………. page 20

***Supplementary Tables***

Supplementary Table 1 ……………………………………. page 21
Supplementary Table 2 ……………………………………. page 23
Supplementary Table 3 ……………………………………. page 25
Supplementary Table 4 ……………………………………. page 26
Supplementary Table 5 ……………………………………. page 27
Supplementary Table 4 ……………………………………. page 27
Supplementary References ……………………………..… page 28

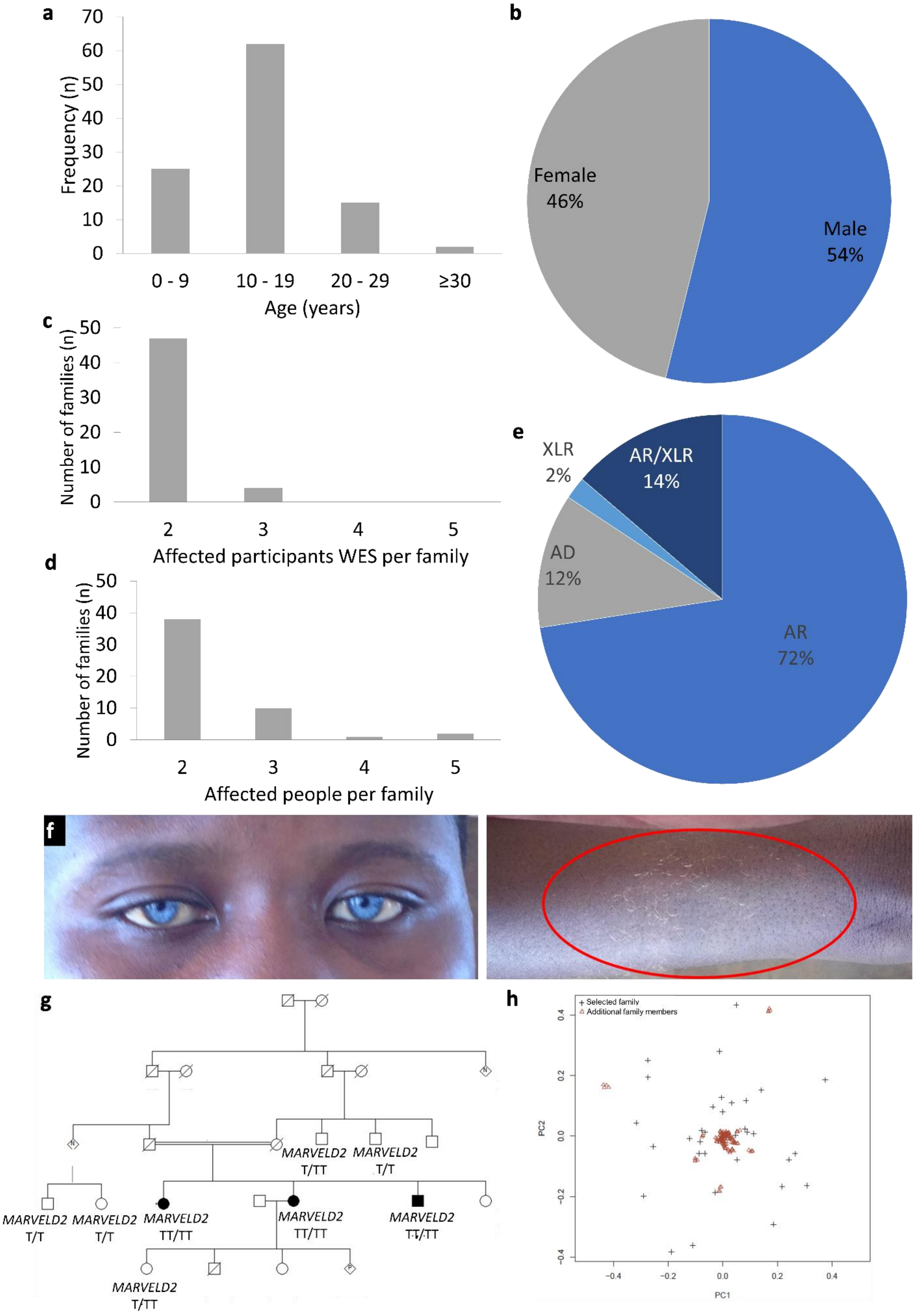


**Supplementary Figure 1. Demographic characterization of patients, pedigree analysis and Waardenburg syndrome clinical features.** (a) Age at ascertainment; and (b) sex

distribution of affected participants. (c) Number of affected family members with WES. (d) Number of affected family members per family. (e) Pie chart of the mode of HI inheritance in the families studied AR = autosomal recessive, AD = autosomal dominant, XLR = X-linked recessive. Characteristic (f) blue eyes and grey hair of a study participant showing clinical signs for Waardenburg syndrome. Informed consent was obtained from the study participants for publishing their images. (g) Pedigree of Fam18, A family with post-lingual HI. TT/TT; T/TT; and T/T denotes homozygote for the *MARVELD2*:c.1058dupT variant, heterozygote, and homozygote wild type, respectively. The black shaded square and circles were used to denote hearing-impaired males and females, respectively. The unshaded squares and circles correspond to hearing males and females. (h) PCA plot projecting family members against the coordinates of the selected family members.

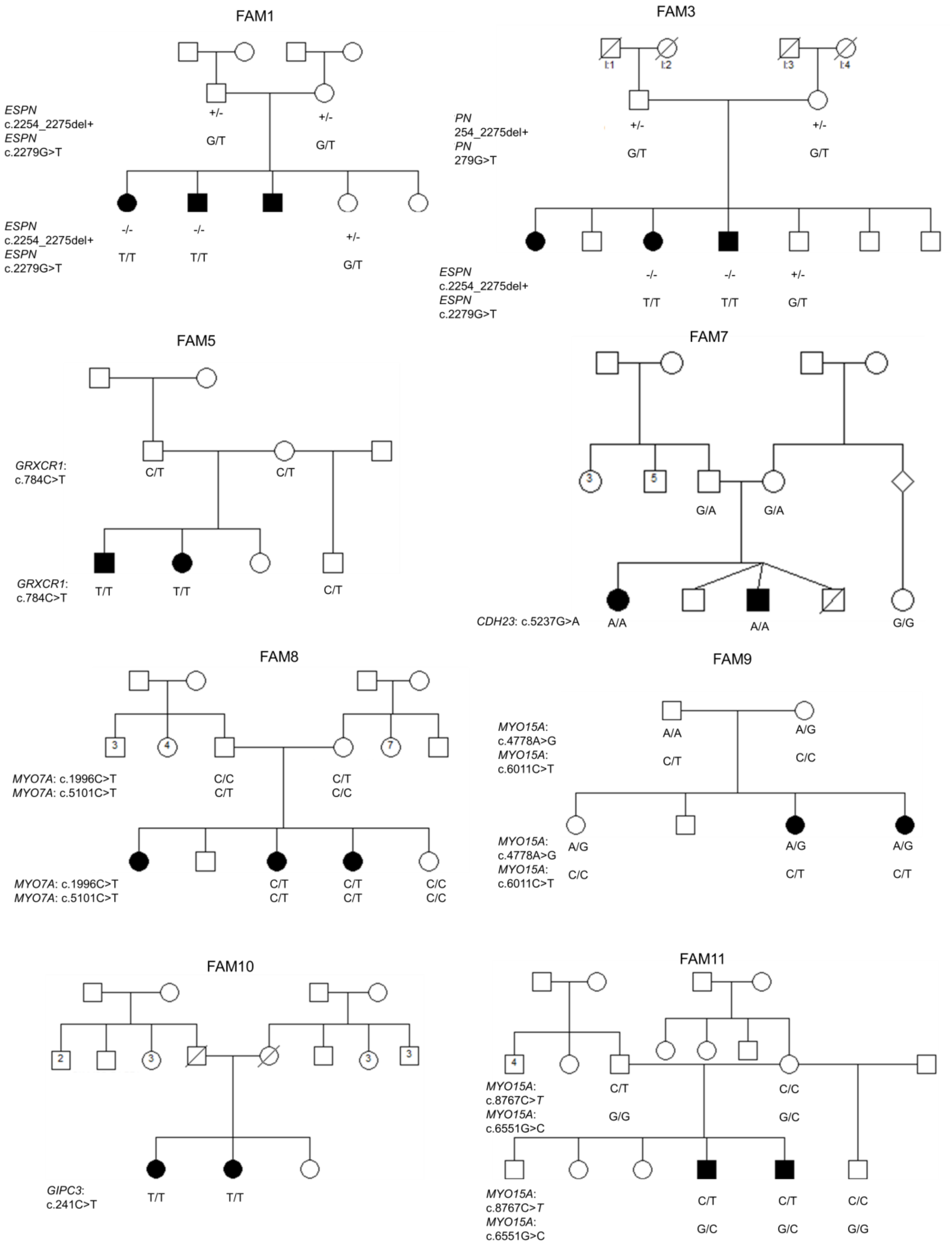


**Supplementary Figure 2:** Pedigree of families (Fam1 – Fam11) with known variants. The segregation of the major causal variants is indicated on the respective pedigrees. The black shaded square and circles were used to denote hearing-impaired males and females, respectively. The unshaded squares and circles correspond to hearing males and females.

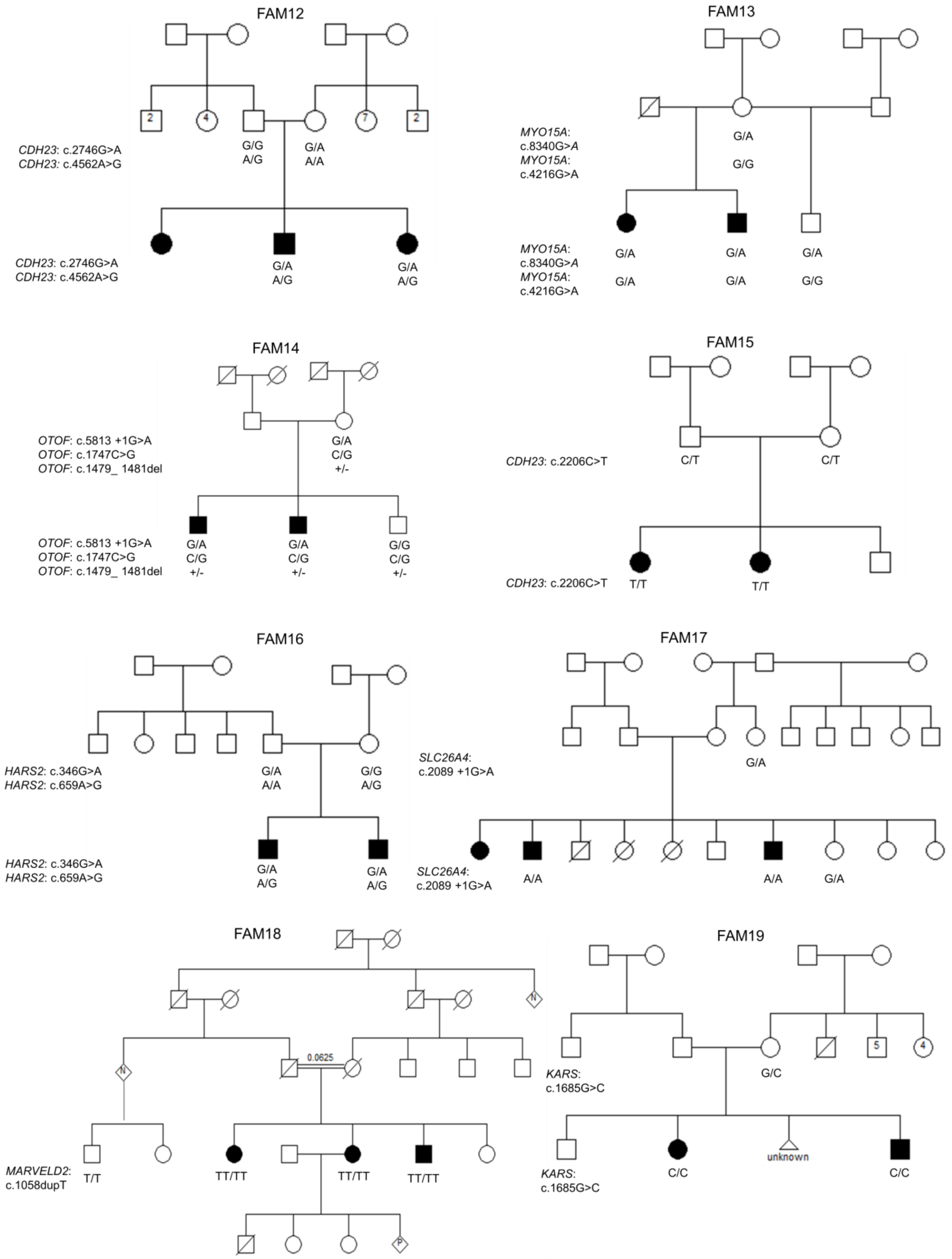


**Supplementary Figure 3:** Pedigree of families (Fam12 – Fam19) with known variants. The segregation of the major causal variants are indicated on the respective pedigrees. The black shaded square and circles were used to denote hearing-impaired males and females, respectively. The unshaded squares and circles correspond to hearing males and females.

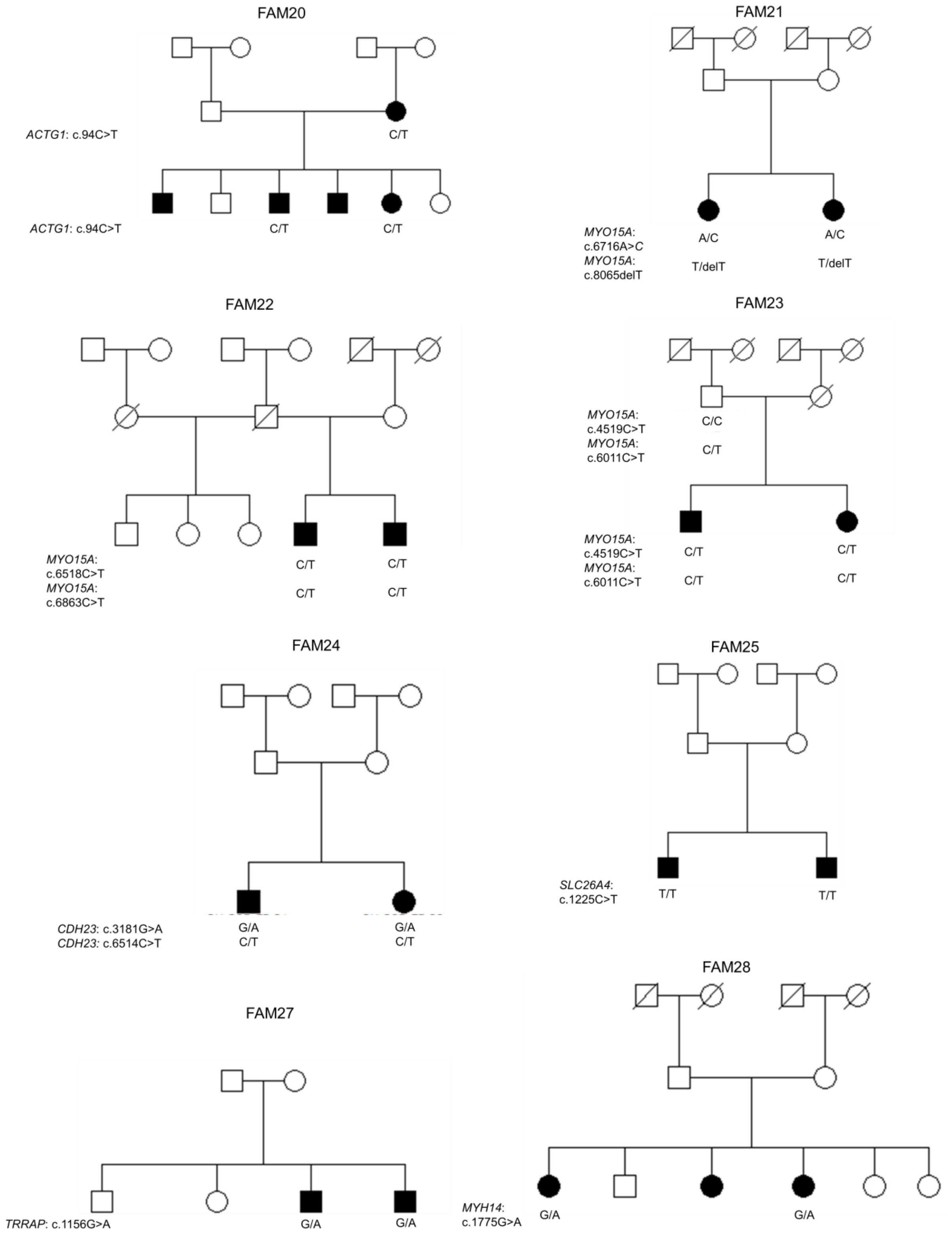


**Supplementary Figure 4:** Pedigree of families (Fam20 – Fam28) with known variants. The segregation of the major causal variants are indicated on the respective pedigrees. The black shaded square and circles were used to denote hearing-impaired males and females, respectively. The unshaded squares and circles correspond to hearing males and females.

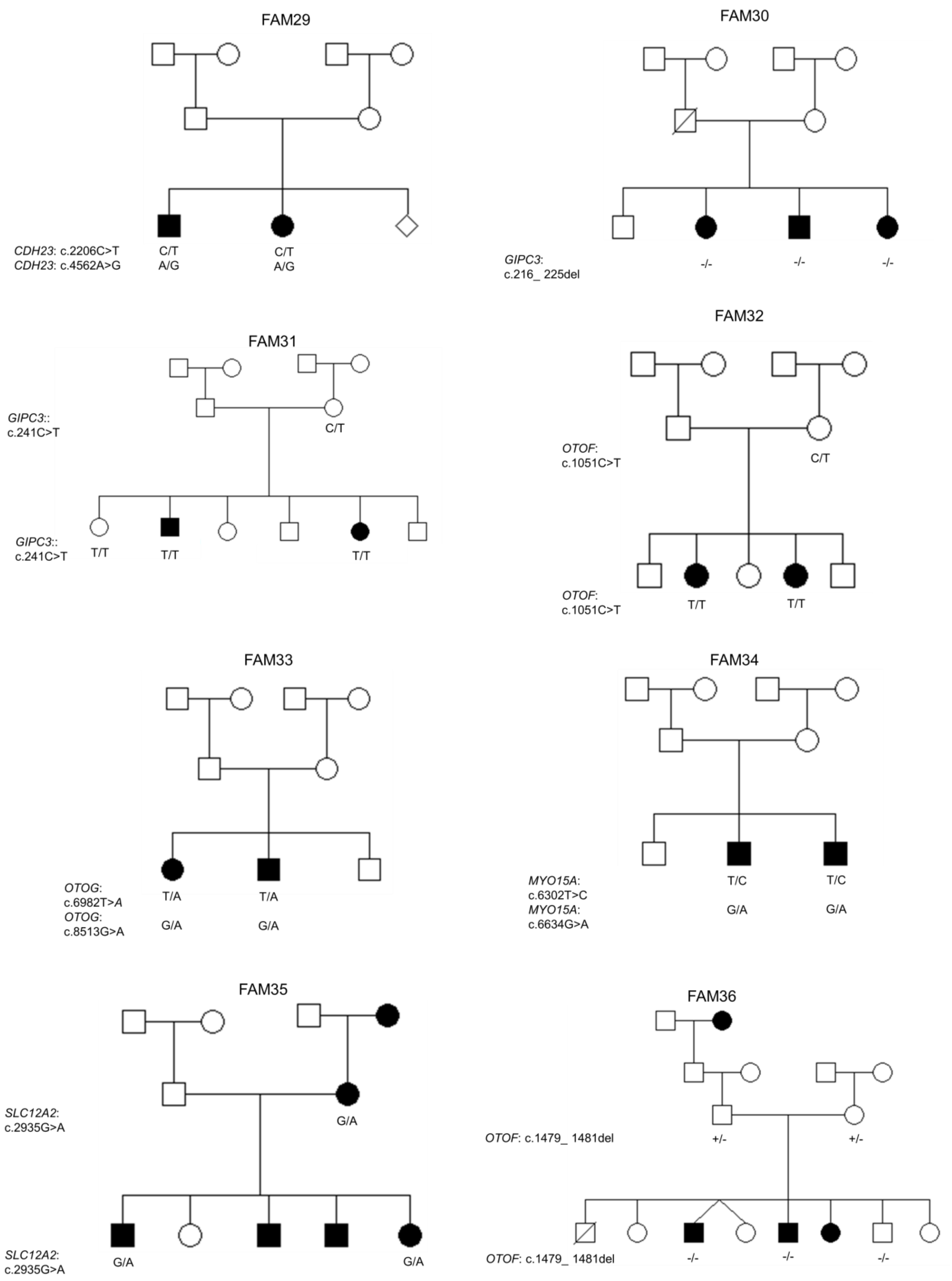


**Supplementary Figure 5:** Pedigree of families (Fam29 – Fam36) with known variants. The segregation of the major causal variants is indicated on the respective pedigrees. The black shaded square and circles were used to denote hearing-impaired males and females, respectively. The unshaded squares and circles correspond to hearing males and females.

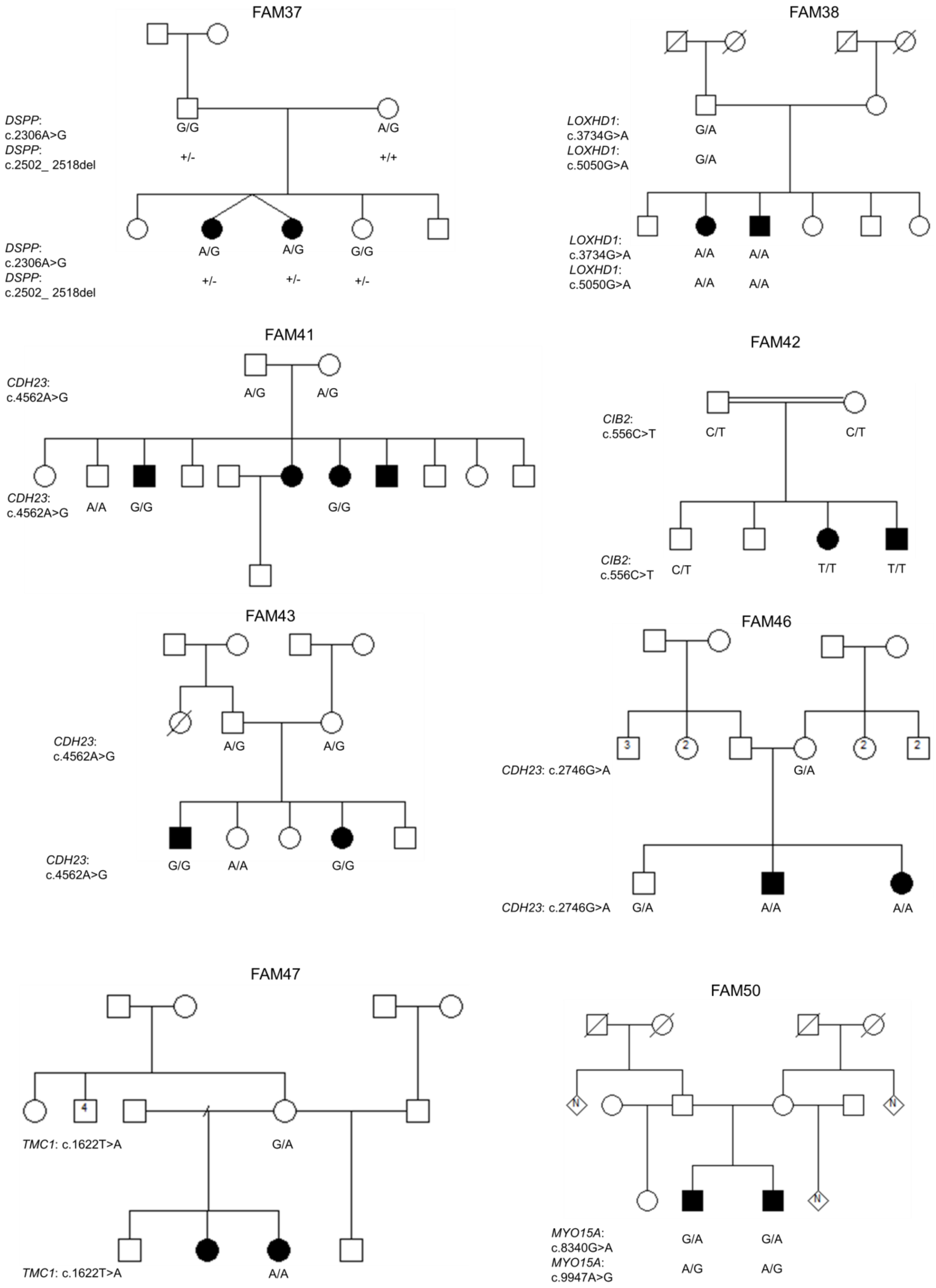


**Supplementary Figure 6:** Pedigree of families (Fam37 – Fam50) with known variants. The segregation of the major causal variants are indicated on the respective pedigrees. The black shaded square and circles were used to denote hearing-impaired males and females, respectively. The unshaded squares and circles correspond to hearing males and females.

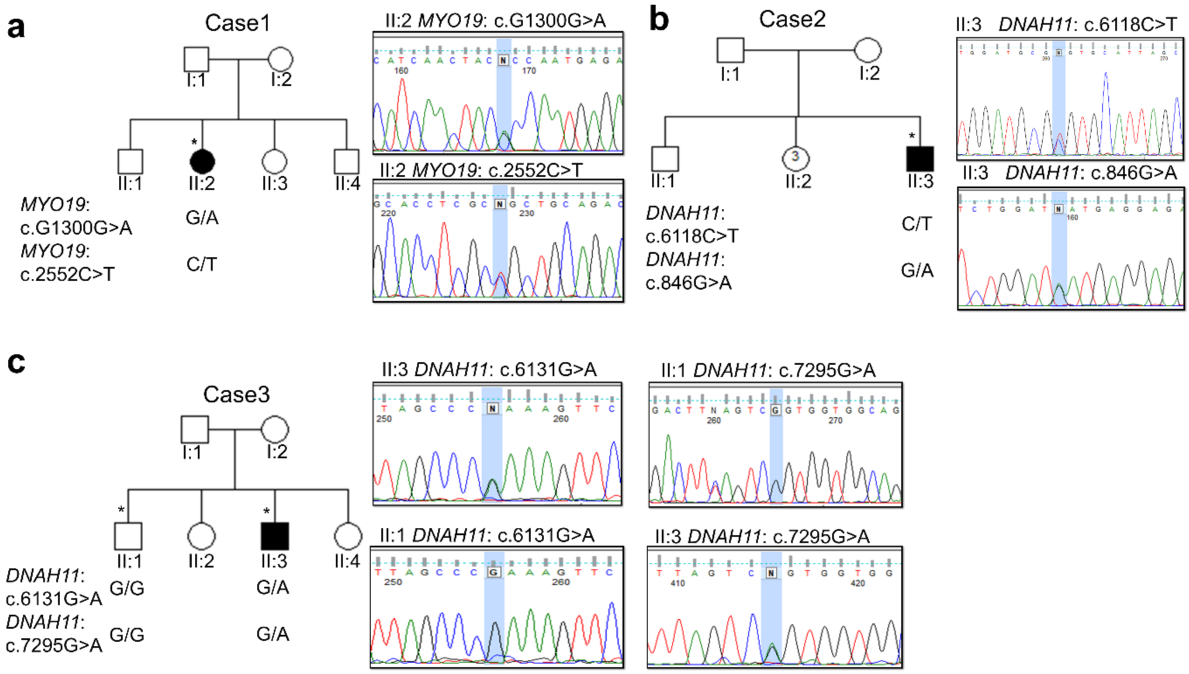


**Supplementary Figure 7: Ghanaian non-familial HI cases with variations in *DNAH11* and *MYO19*.** (a) Ghanaian case with compound heterozygous variants in *MYO19* gene. (b and c) Non-familial Ghanaian cases with *DNAH11* variants. * Individuals whose audiogram was obtained.

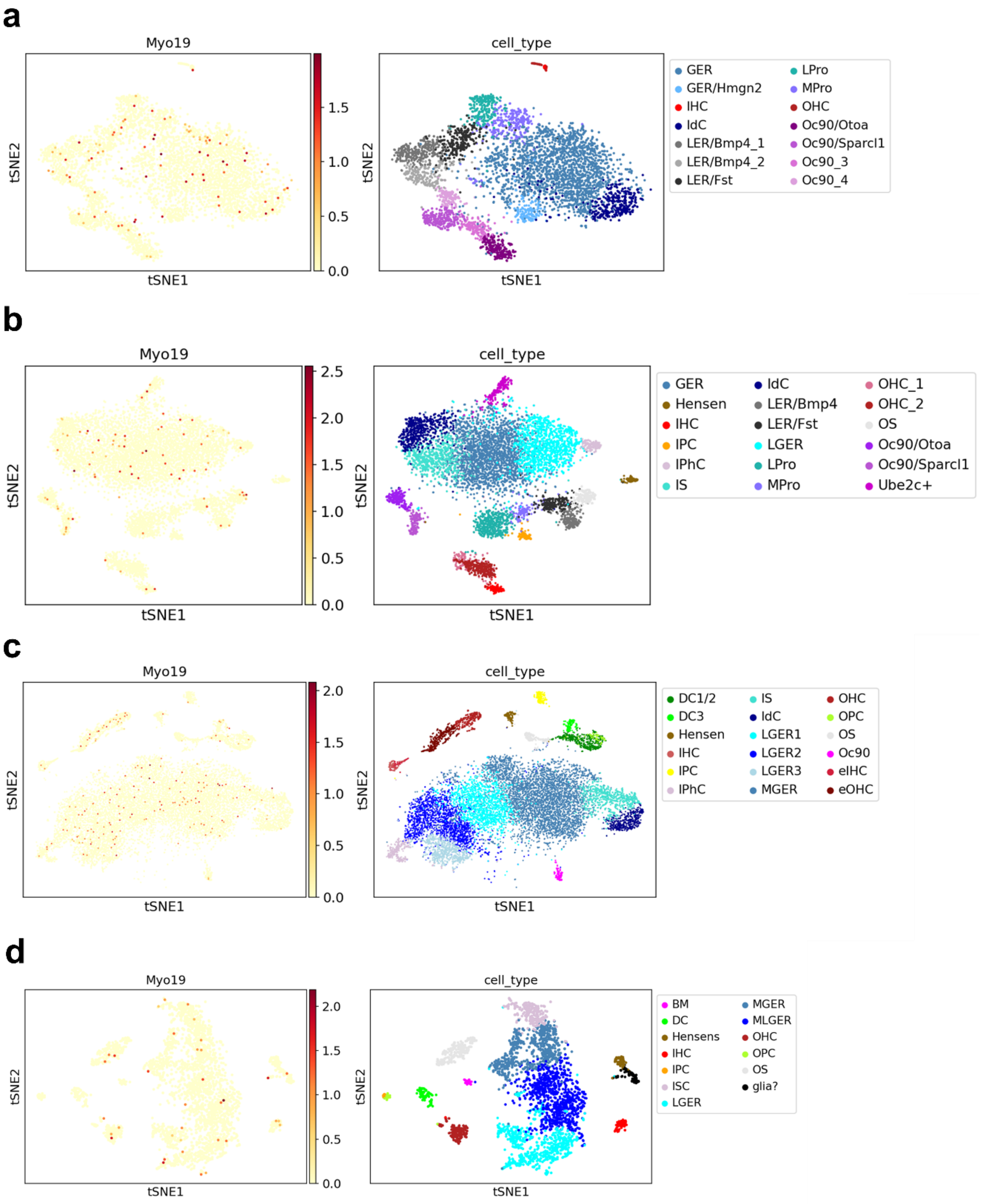


**Supplementary Figure 8. Expression of Myo19 during mouse inner ear development.** Single cell RNA sequencing expression data of the cochlear floor epithelia of CD1 mice is presented in terms of both expression level (left; red = high expression, yellow = low expression) and its expression patterns (right; colors indicate the cell type) [1]. The scale bar represents gene expression based on log transformed, normalized, and scaled for sequencing depth expression data. (a) Myo19 expression during E14; (b) Myo19 expression during E16; (c) Myo19 expression during P1; (d) Myo19 expression during P7. Data were obtained from gEAR (gene Expression Analysis Resource) and plots were generated in its analysis suite.

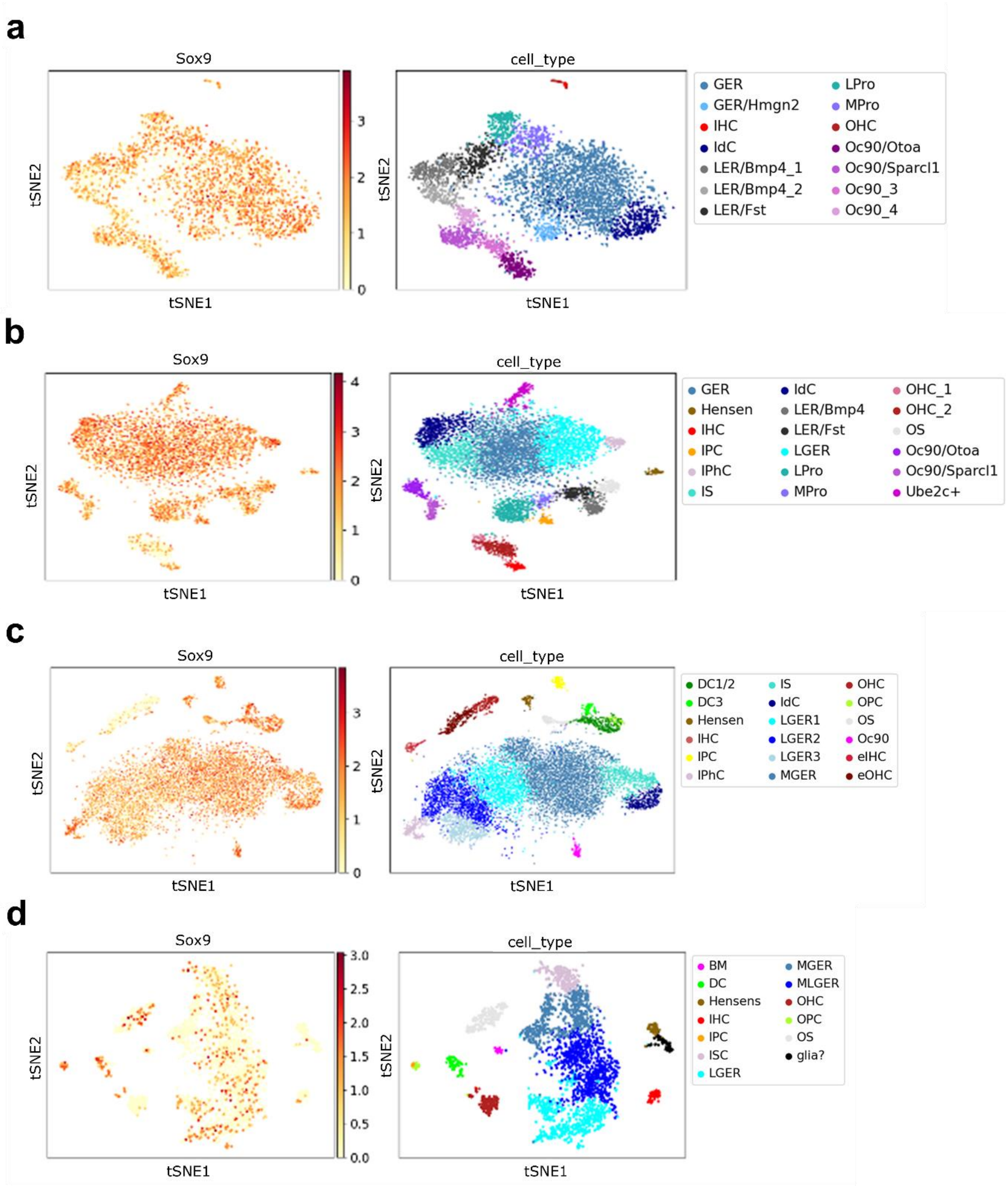


**Supplementary Figure 9. Expression of Sox9 during mouse inner ear development.** Single cell RNA sequencing expression data of the cochlear floor epithelia of CD1 mice is presented in terms of both expression level (left; red = high expression, yellow = low expression) and its expression patterns (right; colors indicate the cell type) [1]. The scale bar represents gene expression based on log-transformed, normalized, and scaled for sequencing depth expression data. (a) Sox9 expression during E14; (b) Sox9 expression during E16; (c) Sox9 expression during P1; (d) Sox9 expression during P7. Data were obtained from gEAR (gene Expression Analysis Resource) and plots were generated in its analysis suite.

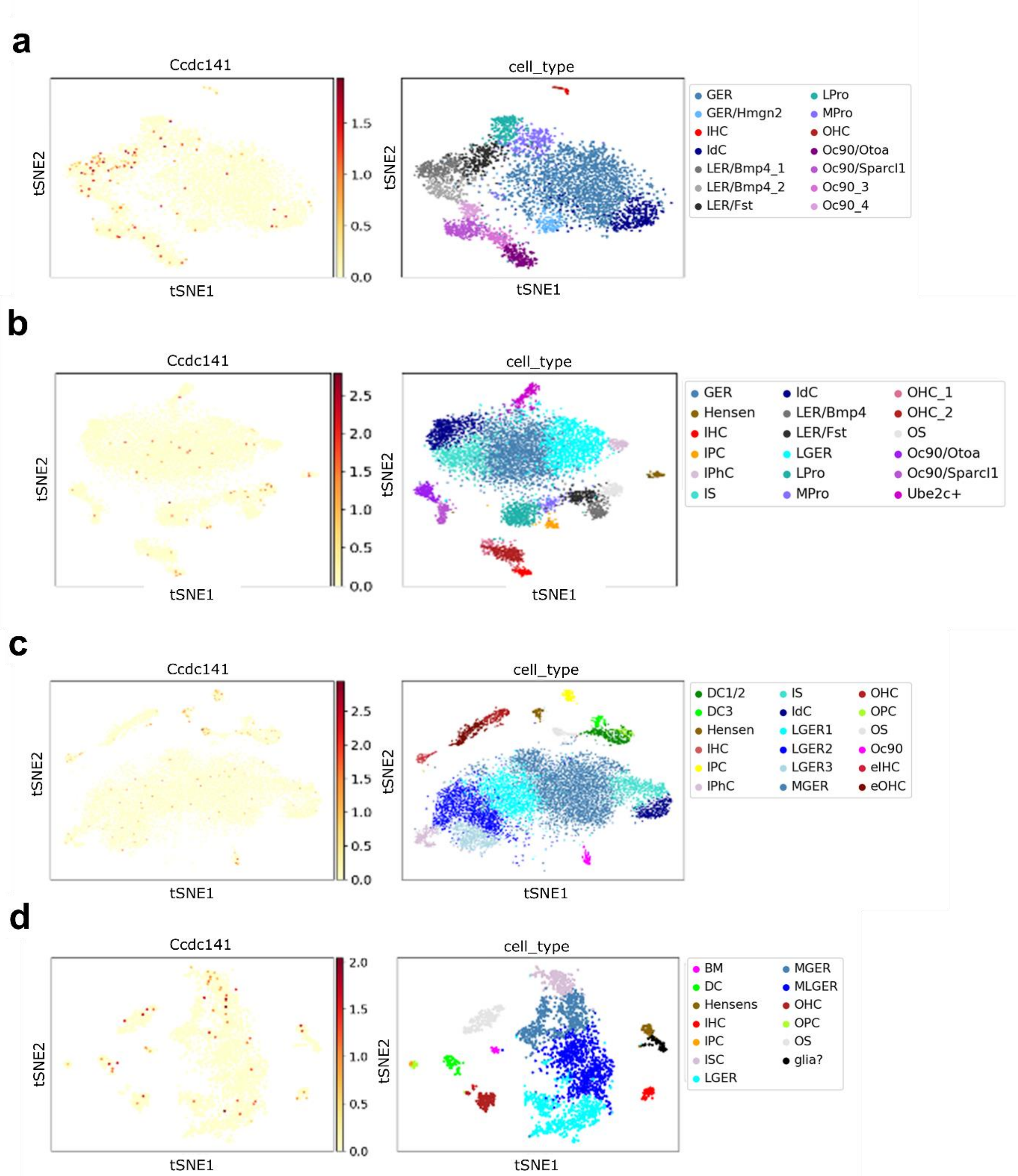


**Supplementary Figure 10**. **Expression of Ccdc141 during mouse inner ear development.** Single cell RNA sequencing expression data of the cochlear floor epithelia of CD1 mice is presented in terms of both expression level (left; red = high expression, yellow = low expression) and its expression patterns (right; colors indicate the cell type) [1]. The scale bar represents gene expression based on log transformed, normalized, and scaled for sequencing depth expression data. (a) Ccdc141 expression during E14; (b) Ccdc141 expression during E16; (c) Ccdc141 expression during P1; (d) Ccdc141 expression during P7. Data were obtained from gEAR (gene Expression Analysis Resource) and plots were generated in its analysis suite.

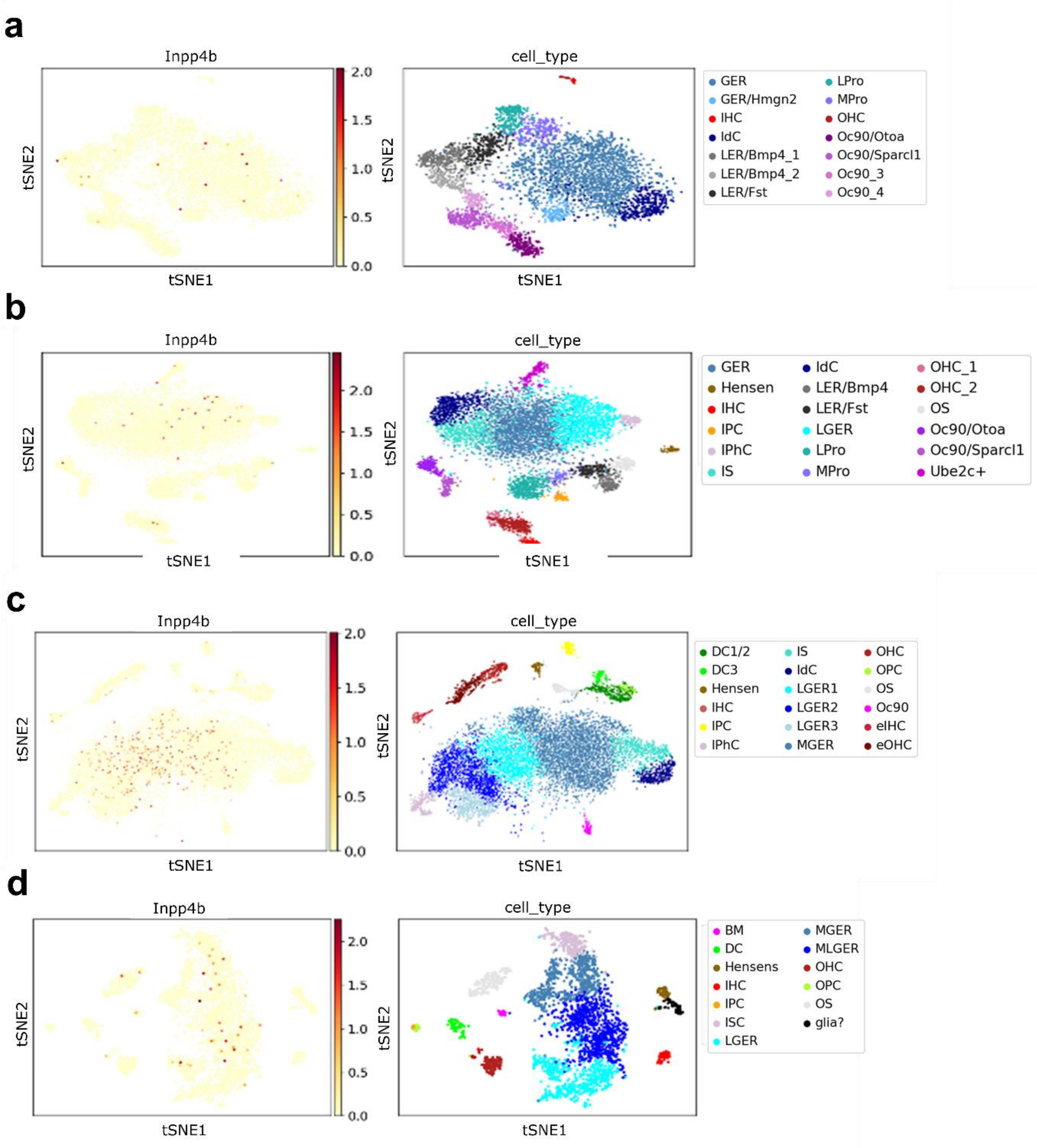


**Supplementary Figure 11. Expression of Inpp4b during mouse inner ear development.** Single cell RNA sequencing expression data of the cochlear floor epithelia of CD1 mice is presented in terms of both expression level (left; red = high expression, yellow = low expression) and its expression patterns (right; colors indicate the cell type) [1]. The scale bar represents gene expression based on log-transformed, normalized, and scaled for sequencing depth expression data. (a) Inpp4b expression during E14; (b) Inpp4b expression during E16; (c) Inpp4b expression during P1; (d) Inpp4b expression during P7. Data were obtained from gEAR (gene Expression Analysis Resource) and plots were generated in its analysis suite.

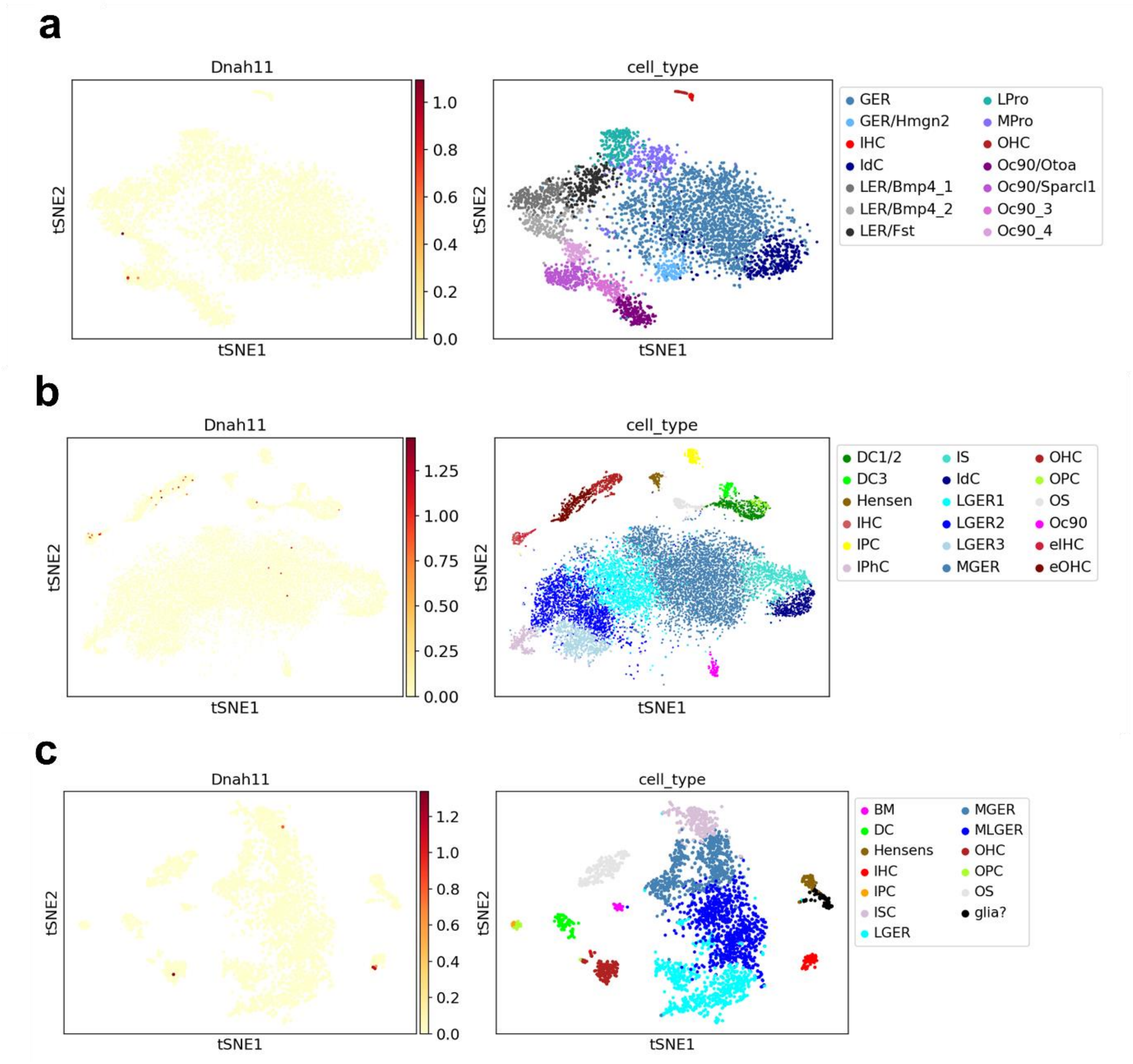


**Supplementary Figure 12. Expression of Dnah11 during mouse inner ear development.** Single cell RNA sequencing expression data of the cochlear floor epithelia of CD1 mice is presented in terms of both expression level (left; red = high expression, yellow = low expression) and its expression patterns (right; colors indicate the cell type) [1]. The scale bar represents gene expression based on log-transformed, normalized, and scaled for sequencing depth expression data. (a) Dnah11 expression during E14; (b) Dnah11 expression during P1; (c) Dnah11 expression during P7. Data were obtained from gEAR (Gene Expression Analysis Resource) and plots were generated in its analysis suite.

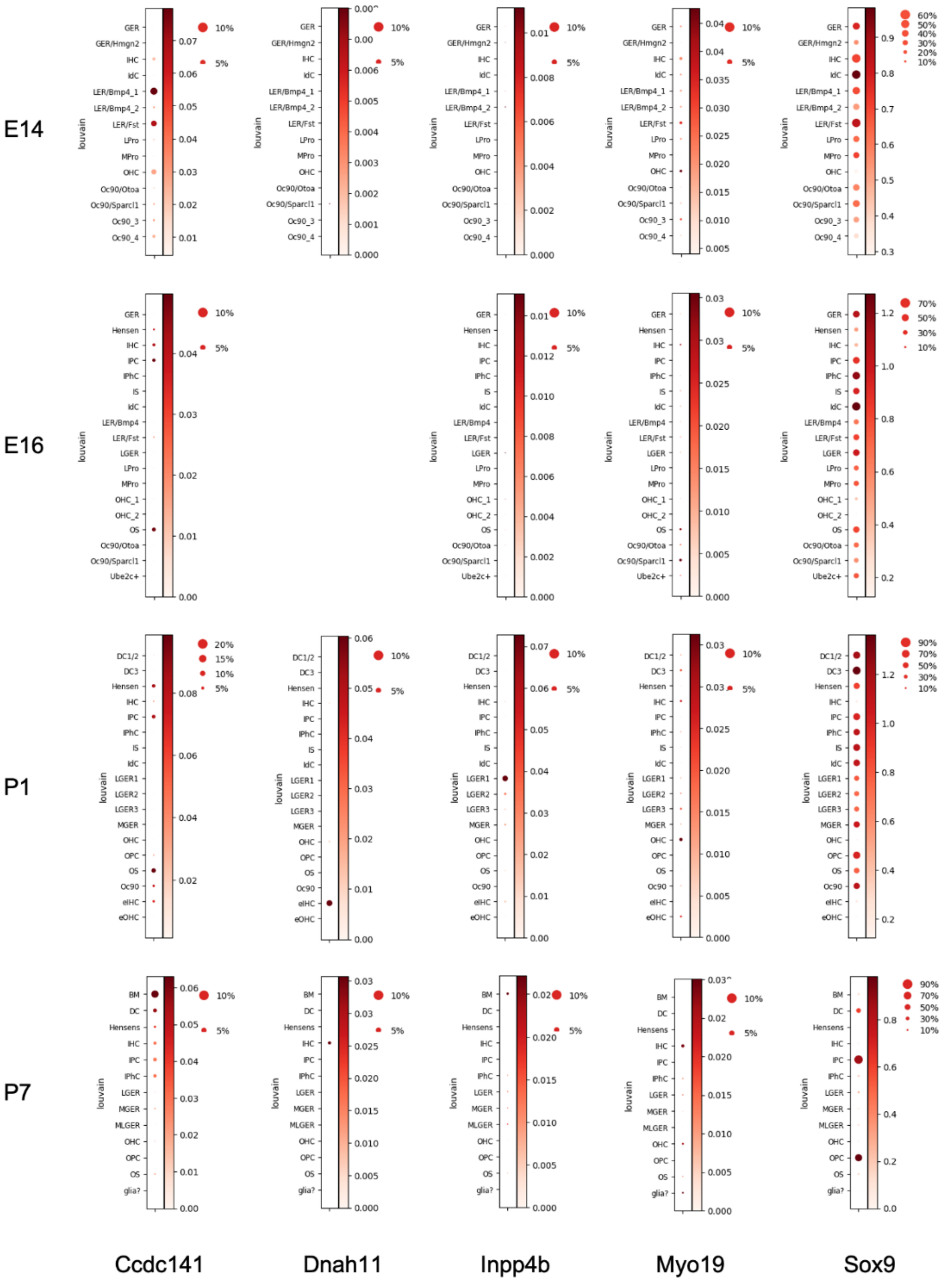


**Supplementary Figure 13. Expression of five novel genes during the development of the mouse inner ear.** Expression levels are presented for each cell type/cluster based on log-transformations and normalization from a single cell RNA sequencing dataset of the cochlear floor epithelia of CD1 mice [1]. The dot size indicates the percentage of cells expressing the gene and the color indicates the intensity of this expression (red = high expression, white = low expression). Notably, though, while Sox9 expression levels decrease in all cells except IPC and OPC, Ccdc141 expression levels increase as development progresses. Data were obtained from gEAR (gene Expression Analysis Resource) and plots were generated in its analysis suite. Pax8 was not available for analysis in this dataset.

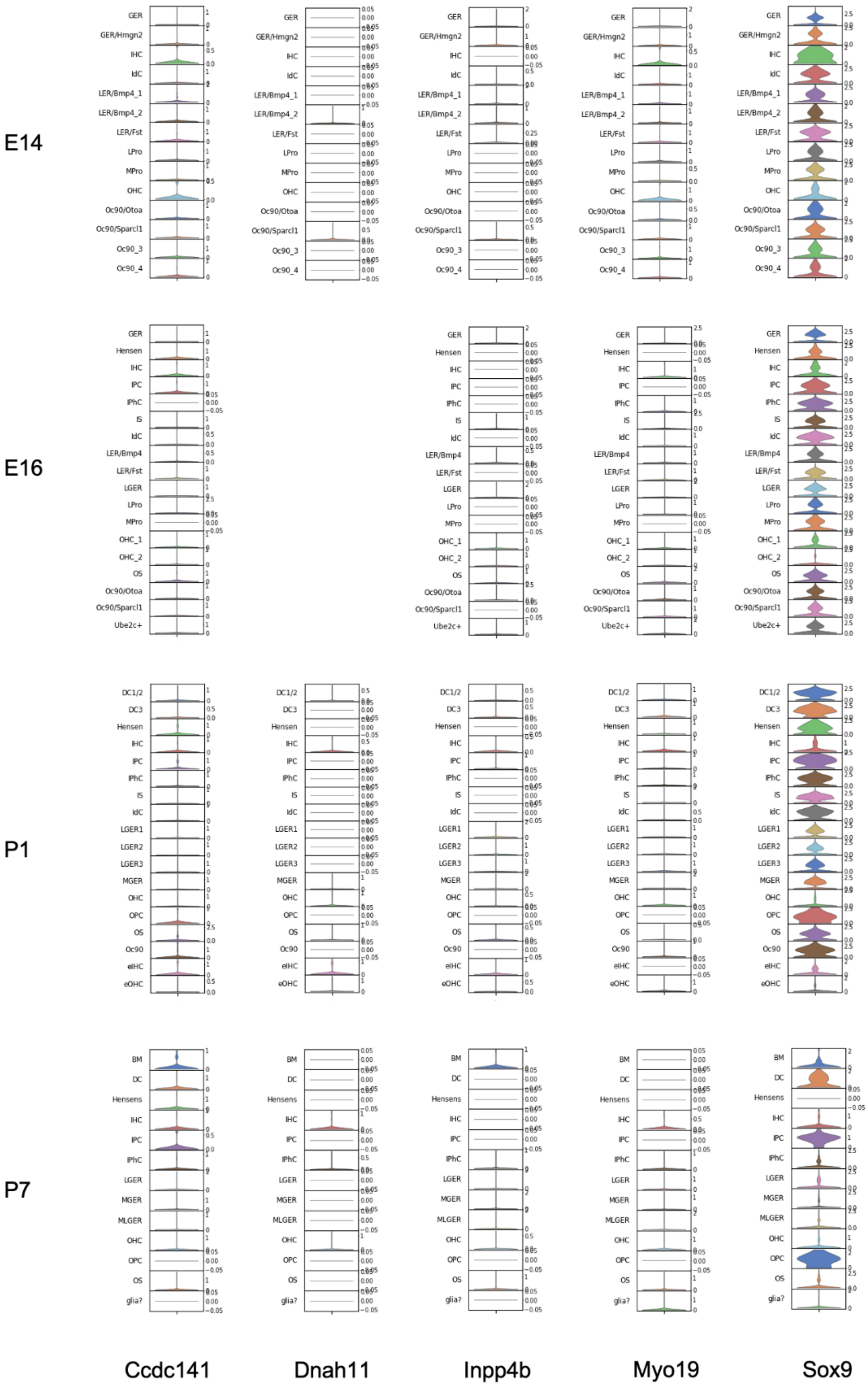


**Supplementary Figure 14. Expression of novel genes during the development of the mouse inner ear**. Data is presented from a single cell RNA sequencing dataset of the cochlear floor epithelia of CD1 mice [1]. Expression is presented as log-transformed and normalized values for each cell type/cluster. The width along the violin plot indicates the frequency of the different intensities. Though Sox9 has a much greater expression overall, it decreases in expression as development progresses whereas other genes such as Ccdc141 increase in expression. Data were obtained from gEAR (gene Expression Analysis Resource) and plots were generated in its analysis suite. Pax8 was not available for analysis in this dataset.

*Abbreviations* [1]*:*

*Developing Supporting Cells:* BM: Basilar Membrane Cells; DC: Dieter Cells; DC1/2: Dieter Cells from rows 1 and 2; DC3: Dieter Cells from row 3; GER: Greater Epithelial Range; GER/Hmgn2: Greater Epithelial Range expressing Hmgn2; Glia: Glial Cells; Hensen: Hensen Cells; IdC: Interdental Cells; IPC: Inner Pillar Cells; IPhC: Inner Phalangeal Cells; IS: Inner Sulcus Cells; ISC: Inner Sulcus Cells; LER/Bmp4_1: Lesser Epithelial Ridge Cells Expressing Bmp4; LER/Bmp4_2: Lesser Epithelial Ridge Cells Expressing Bmp4; LER/Fst: Lesser Epithelial Ridge Cells Expressing Fst; Oc90/Otoa: Cells expressing Oc90 and Otoa; Oc90/Sparcl1: Cells expressing Oc90 and Sparcl1; Oc90_3: Cells expressing Oc90; Oc90_4: Cells expressing Oc90; OPC: Outer Pillar Cells; OS: Outer Sulcus Cells;

*Developing Prosensory Cells:* LPro: Lateral Prosensory Cells; MPro: Medial Prosensory Cells.

*Developing Greater Epithelial Ridge Cells:* LGER: Lateral Great Epithelial Ridge Cells; LGER1: Lateral Great Epithelial Ridge Cells, group 1; LGER2: Lateral Great Epithelial Ridge Cells, group 2; LGER3: Lateral Great Epithelial Ridge Cells, group 3; MGER: Medial Greater Epithelial Ridge Cells; MLGER: Medial Lateral Greater Epithelial Ridge Cells;

*Developing Sensory Cells:* elHC: Less mature developing inner hair cells; eOHC: Less mature developing outer hair cells; IHC: Inner Hair Cells; OHC: Outer Hair Cells; OHC_1: More mature developing outer hair cells; OHC_2: Less mature developing outer hair cells;

*Uncategorized:* Ube2c+: Unannotated cells expressing Ube2c+

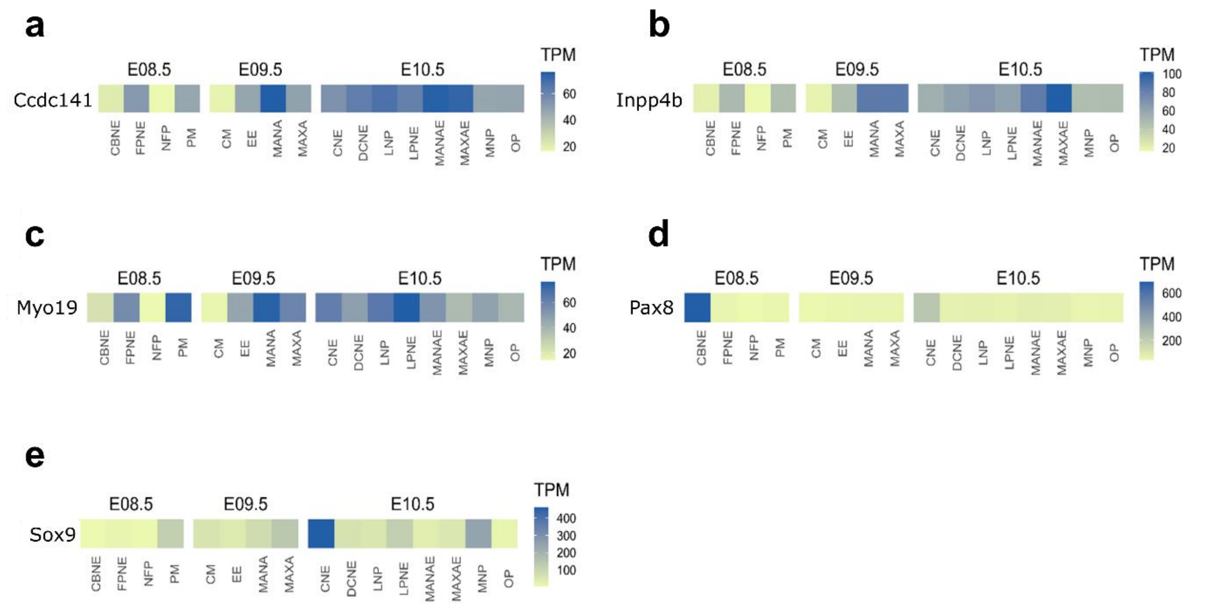


**Supplementary Figure 15. Gene expression of novel candidate genes in various craniofacial tissues during early mouse development.** RNA sequencing data from various mouse craniofacial tissues during three developmental stages: E8.5, E9.5, and E10.5, is presented here [2]. Expression is presented as transcript per million (TPM) values which show that the greatest expression of Ccdc141, Inpp4b, Myo19, and Sox9 occurs during E10.5, whereas the greatest expression of Pax8 occurs during E8.5. Dnah11 was not present in this dataset to interrogate. Heatmaps were generated using R.

*Craniofacial Tissue Codes*: CBNE: Caudal Brain Neural Epithelium; CM: Cranial Mesenchyme; CNE: Central Neural Epithelium; DCNE: Dorsal Control Neural Epithelium; EE: Epidermal Ectoderm; FPNE: Floor Plate Neural Epithelium; LNP: Lateral Nasal Prominence; LPNE: Lateral Prominence Neural Epithelium; MANA: Mandibular Arch; MANAE: Mandibular Arch Epidermal Ectoderm; MAXA: Maxillary Arch; MAXAE: Maxillary Arch Epidermal Ectoderm; MNP: Medial Nasal Prominence; NFP: Non-Floor Plate Neural Epithelium; OP: Olfactory Pit; PM: Paraxial Mesoderm.

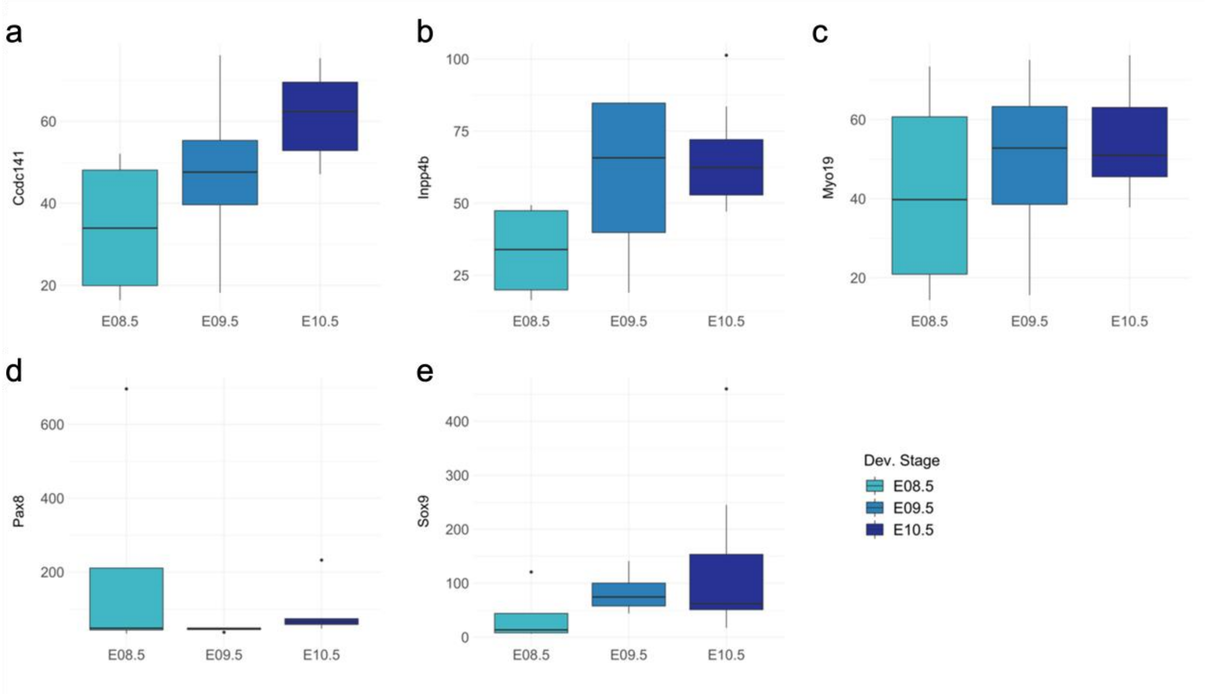


**Supplementary Figure 16**. **Average expression of novel candidate genes in craniofacial tissues during mouse development.** RNA sequencing data sourced from various mouse craniofacial tissues during three developmental stages: E8.5, E9.5, and E10.5, is presented here [2]. Expression is presented as box plots based on TPM values. While Ccdc141, Inpp4b, Myo19, and Sox9 all exhibit their highest levels of expression in E10.5, Pax8 has its greatest expression during E8.5. Dnah11 was not present in this dataset to interrogate. Data was plotted using ggplot2 in R.

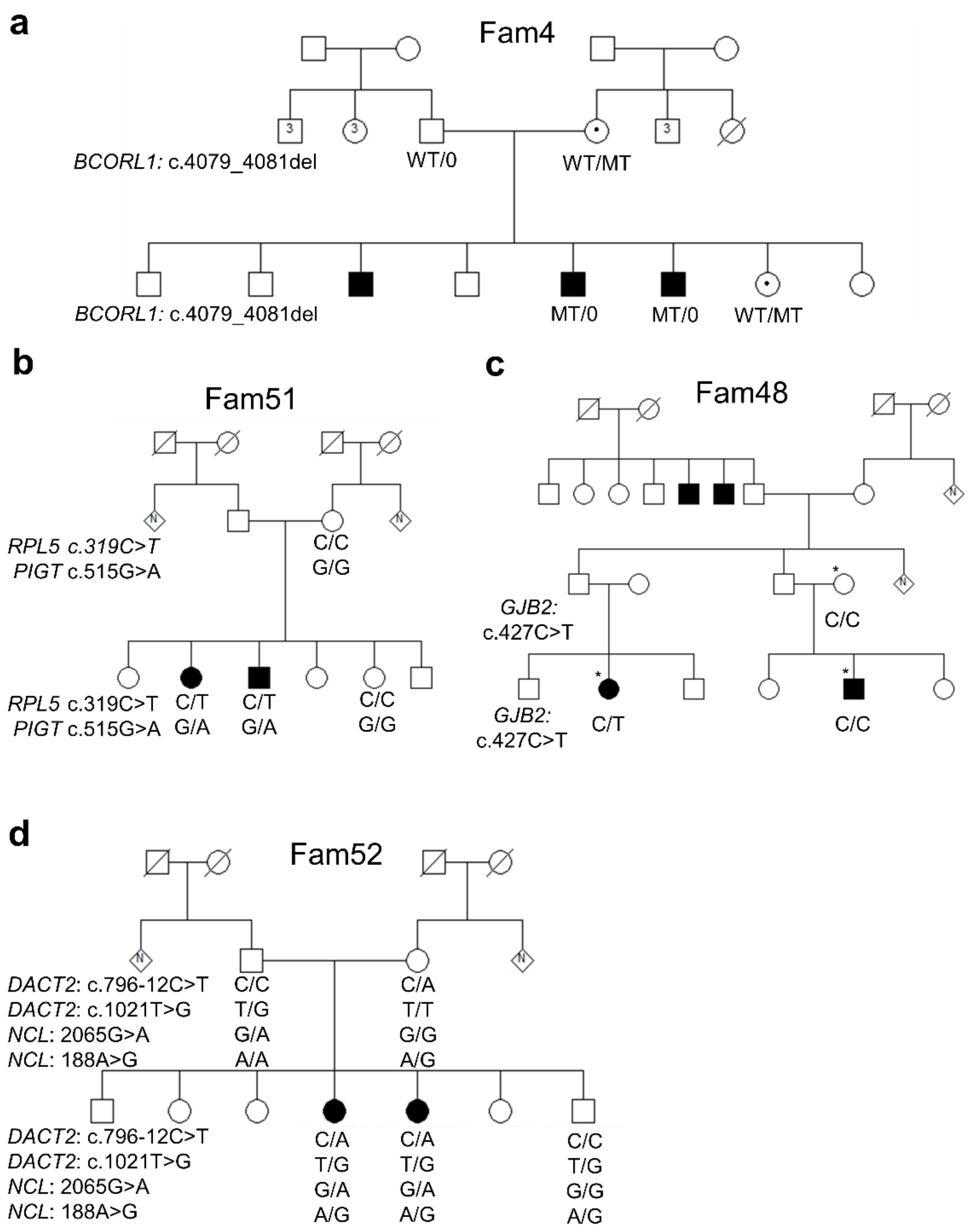


**Supplementary Figure 17:** Pedigrees of unsolved families. The segregation of variants found is shown in the respective families, (a) Fam 4; (b) Fam 51, (c) Fam48 (d) Fam 52, WT/0 represents hemizygous wild type, MT/0 represents hemizygous mutant (deletion), and WT/MT represents heterogenous wild type/mutant (deletion). The black shaded square and circles were used to denote hearing-impaired males and females, respectively. The unshaded squares and circles correspond to hearing males and females. * Individuals whose audiogram was obtained.

**Supplementary Table 1: Variants in Known Genes Identified in *GJB2* negative Ghanaian Families Using Whole Exome Sequencing**

| Gene | NM numbers | Nucleotide change | Protein change | Rs-number | Affected families | gnomAD _AF | gnomAD_ AFR_AF | TOPMed | CADD_ Score | ACMG-AMP classification | Reference |
|---|---|---|---|---|---|---|---|---|---|---|---|
| *CDH23* | NM_022124.5 | c.2206C>T | p.(R736X) | rs1230303971 | 2 | 8.03E-06 | 0.00E+00 | 7.56E-06 | 42 | P | C [3] |
| *CDH23* | NM_022124.5 | c.5237G>A | p.(R1746Q) | rs111033270 | 1 | 6.82E-05 | 6.46E-05 | 1.81E-04 | 27.7 | P | C [4, 5] |
| *CDH23* | NM_022124.5 | c.2746G>A | p.(D916N) | rs1318444606 | 2 | - | - | 1.13E-05 | 28.5 | LP | € |
| *CDH23* | NM_022124.5 | c.4562A>G | p.(N1521S) | rs780987516 | 4 | 1.20E-05 | 6.46E-05 | 5.29E-05 | 23.3 | LP | € |
| *CDH23* | NM_022124.5 | c.3181G>A | p.(E1061K) | rs1060499793 | 1 | - | - | - | 31 | LP | € |
| *CDH23* | NM_022124.5 | c.6514C>T | p.(P2172S) | - | 1 | - | - | - | 21.4 | LP | X |
| *MYO15A* | NM_016239.3 | c.4778A>G | p.(E1593G) | - | 1 | - | - | - | 35 | LP | X |
| *MYO15A* | NM_016239.3 | c.6011C>T | p.(P2004L) | rs756172590 | 2 | 9.25E-06 | 0.00E+00 | 3.78E-06 | 29.4 | LP | € |
| *MYO15A* | NM_016239.3 | c.8767C>T | p.(R2923X) | rs373462792 | 1 | 0.00E+00 | 0.00E+00 | 4.53E-05 | 37 | P | € |
| *MYO15A* | NM_016239.3 | c.6551G>C | p.(C2184S) | - | 1 | - | - | - | 25.2 | LP | X |
| *MYO15A* | NM_016239.3 | c.8340G>A | p.(T2780T) | rs878853228 | 2 | 8.03E-06 | 0.00E+00 | 3.78E-06 | 22.8 | VUS | € |
| *MYO15A* | NM_016239.3 | c.4216G>A | p.(E1406K) | rs759810756 | 1 | 8.03E-06 | 0.00E+00 | 3.78E-06 | 31 | LP | C [6] |
| *MYO15A* | NM_016239.3 | c.1196A>G | p.(Y399C) | rs368682932 | 1 | 6.42E-05 | 7.12E-04 | 3.66E-04 | 22.9 | VUS | € |
| *MYO15A* | NM_016239.3 | c.6716A>C | p.(H2239P) | rs760577812 | 1 | 5.10E-05 | 0.00E+00 | 7.56E-06 | 25.3 | LP | € |
| *MYO15A* | NM_016239.3 | c.8065delT | p.(W2689Gfs*49) | rs1567654885 | 1 | 4.02E-06 | 0.00E+00 | - | 28.6 | P | € |
| *MYO15A* | NM_016239.3 | c.6518C>T | p.(S2173F) | - | 1 | - | - | - | 29.1 | LP | X |
| *MYO15A* | NM_016239.3 | c.6863C>T | p.(S2288L) | rs886052676 | 1 | 8.52E-06 | 0.00E+00 | 1.89E-05 | 25.3 | VUS | € |
| *MYO15A* | NM_016239.3 | c.4519C>T | p.(R1507X) | rs549138385 | 1 | 4.01E-06 | 0.00E+00 | 1.89E-05 | 40 | P | C [7] |
| *MYO15A* | NM_016239.3 | c.6302T>C | p.(L2101P) | rs201908493 | 1 | 2.00E-05 | 8.00E-04 | 2.08E-04 | 27.4 | LP | € |
| *MYO15A* | NM_016239.3 | c.6634G>A | p.(E2212K) | rs371352836 | 1 | 2.41E-05 | 0.00E+00 | 2.27E-05 | 28.4 | LP | € |

| | | | | | | | | | | | |
|---|---|---|---|---|---|---|---|---|---|---|---|
| *GIPC3* | NM_133261.2 | c.241C>T | p.(L81F) | rs1487341857 | 2 | - | - | 1.89E-05 | 25.6 | VUS | € |
| *GIPC3* | NM_133261.2 | c.216_ 225del | p.(P73Ffs*21) | - | 1 | - | - | - | - | P | X |
| *OTOF* | NM_194248.2 | c.1051C>T | p.(Q351X) | rs1558492758 | 1 | - | - | - | 37 | P | € |
| *OTOF* | NM_194248.2 | c.5813 +1G>A | | | 1 | - | - | - | 34 | P | X |
| *OTOF* | NM_194248.2 | c.1747C>G | p.(P583A) | rs756894987 | 1 | 4.42E-05 | 0.00E+00 | 5.67E-05 | - | VUS | € |
| *OTOF* | NM_194248.2 | c.1479_ 1481del | p.(K493_ R494delinsN) | - | 2 | - | - | - | - | LP | X |
| *ESPN* | NM_031475.2 | c.2254_2275del+ c.2279G>T[#] | p.(E731Cfs*6) + p.(R760L) | rs371306949 | 2 | 4.36E-05 | 3.88E-04 | 2.64E-05 | - | P | € |
| *SLC26A4* | NM_000441.1 | c.2089 +1G>A | - | rs727503430 | 1 | 1.35E-05 | 1.39E-04 | 3.40E-05 | 35 | P | C [8, 9] |
| *SLC26A4* | NM_000441.1 | c.1225C>T | p.(R409C) | rs147952620 | 1 | 1.59E-05 | 6.15E-05 | | 29.8 | LP | C [9, 10] |
| *GRXCR1* | NM_001080476.2 | c.784C>T | p.(R262X) | rs761349153 | 1 | 3.21E-05 | 0 | 2.64E-05 | 45 | P | C [11] |
| *MYO7A* | NM_000260.3 | c.1996C>T | p.(R666X) | rs121965085 | 1 | 1.63E-05 | 0 | 1.13E-05 | 38 | P | C [12, 13] |
| *MYO7A* | NM_000260.3 | c.5101C>T | p.(R1701X) | rs111033182 | 1 | 1.11E-05 | 0 | 3.78E-06 | 39 | P | C [14, 15] |
| *HARS2* | NM_001278731.1 | c.346G>A | p.(A116T) | rs1312606802 | 1 | - | - | 3.78E-06 | 29.2 | LP | € |
| *HARS2* | NM_001278731.1 | c.659A>G | p.(Y220C) | rs746757469 | 1 | 1.19E-05 | 0 | 1.89E-05 | 28.6 | LP | € |
| *MARVELD2* | NM_001244734.1 | c.1058dupT | p.(V354Sfs*5) | | 1 | - | - | - | 16.48 | P | X |
| *KARS* | NM_001130089.1 | c.1685G>C | p.(C562S) | rs1156833108 | 1 | 3.98E-06 | 0 | - | 28.8 | LP | € |
| *ACTG1* | NM_001614.3 | c.94C>T | p.(P32S) | rs1598551290 | 1 | - | - | - | 0.475 | VUS | C [16] |
| *TRRAP* | NM_003496.3 | c.1156G>A | p.(V386I) | - | 1 | - | - | - | 24.5 | VUS | X |
| *MYH14* | NM_001145809.1 | c.1775G>A | p.(R592Q) | rs1445498283 | 1 | 4.02E-06 | 0 | 1.89E-05 | 23.9 | VUS | € |
| *OTOG* | NM_001277269.1 | c.6982T>A | p.(S2328T) | rs1274985459 | 1 | 6.63E-06 | 0 | 3.78E-06 | 17.83 | VUS | € |
| *OTOG* | NM_001277269.1 | c.8513G>A | p.(R2838H) | rs544815967 | 1 | 1.41E-04 | 1.93E-03 | 5.40E-04 | 29.7 | VUS | € |
| *DSPP* | NM_014208.3 | c.2306A>G | p.(D769G) | rs370931212 | 1 | 1.12E-04 | 1.50E-03 | 5.82E-04 | 21.4 | VUS | € |

| | | | | | | | | | | | |
|---|---|---|---|---|---|---|---|---|---|---|---|
| *DSPP* | NM_014208.3 | c.2502_ 2518del | p.(D834Efs*2) | - | 1 | - | - | - | - | NA | X |
| *LOXHD1* | NM_144612.6 | c.3734G>A | p.(G1245D) | - | 1 | - | - | - | 25.3 | VUS | X |
| *LOXHD1* | NM_144612.6 | c.5050G>A | p.(A1684T) | rs376122149 | 1 | 1.52E-04 | 2.19E-03 | 6.88E-04 | 20.8 | VUS | € |
| *CIB2* | NM_006383.3 | c.556C>T | p.(R186W) | rs370359511 | 1 | 3.19E-05 | 4.31E-04 | 2.80E-04 | 32 | LP | C [17, 18, 19] |
| *TMC1* | NM_138691.2 | c.1622T>A | p.(I541N) | - | 1 | - | - | - | 27.5 | VUS | X |
| *KCNT1* | | c.1769+9G>A | | rs758402819 | 1 | 2.9 E-05 | 0.0 | 2.6 E-05 | 3.42 | LB | € |
| *KCNT1* | | c.3256G>C | p.G1086R | | 1 | 2.67 E-04 | 3.34 E-03 | 1.25E-03 | 14.69 | VUS | X |
| *SLC12A2* | | c.2935G>A | p.(E979K) | rs1581138934 | 1 | - | - | - | 23 | LP | C [20] |

Rs-number = Reference number, genotype, gnomAD_AF = global population allele frequency on the Genome Aggregation Database (gnomAD), gnomAD_AFR_AF = African population allele frequency on the Genome Aggregation Database (gnomAD), ACMG-AMP = American College of Medical Genetics guidline for classifying variants as pathogenic (P), likely pathogenic (LP), uncertain significance (VUS), likely benign (LB), or benign (B); NA: Not Applicable. The criteria for the ACMG-AMP classifications are summarized in Supplementary Table 2. X = likely novel variant, € = variants present on the database that were newly associated with HI, C = previously reported HI variant. [#]Variants are both on the same haplotype, the missense variant (R760L) is predicted to be at position 753 as part of the frameshift sequence.

## Supplementary Table 2: ACMG-AMP classification of variants

| Candidate Gene | Nucleotide change | Protein change | ACMG criteria* | Verdict* |
|---|---|---|---|---|
| *ACTG1* | c.94C>T | p.(P32S) | PS1_S, PM2, PP1_P, PP3 | LP |
| *CDH23* | c.5237G>A | p.(R1746Q) (splicing) | PS1_S, PM2_P, PP1_P | LP |
| *CDH23* | c.2746G>A | p.(D916N) | PM1_M, PM2_P, PP1_P, PP3 | LP |
| *CDH23* | c.4562A>G | p.(N1521S) | PM1_M, BS1_P, PP1_P, PP3, PM3_P | LP |
| *CDH23* | c.2206C>T | p.(R736X) | PVS1_VS, PM2, PP1_P | LP |
| *CDH23* | c.3181G>A | p.(E1061K) | PM1_M, PM2, PP1_P, PM3_P | LP |
| *CDH23* | c.6514C>T | p.(P2172S) | PM1_M, PM2_P, PP1_P, PP3 | LP |
| *CIB2* | c.556C>T | p.(R186W) | PM1_M, PM2_P, PP1_P | VUS |
| *ESPN* | c.2279G>T | p.(E731Cfs*6) + p.(R760L) | PVS1_VS, PM2_P, PP1_P | LP |
| *GIPC3* | c.241C>T | p.(L81F) | PM2_P, PP1_P, PP3 | VUS |
| *GIPC3* | c.216_ 225del | p.(P73Ffs*21) | PVS1_VS, PM2_P, PP1_P | LP |
| *GRXCR1* | c.784C>T | p.(R262X) | PVS1_S, PM2_P, PP1_P | LP |
| *HARS2* | c.346G>A | p.(A116T) | PM1_M, PM2_P, PP1_P | VUS |
| *HARS2* | c.659A>G | p.(Y220C) | PM2_P, PP1_P, PP3 | VUS |
| *KARS* | c.1685G>C | p.(C562S) | PM2, PP1_P, PP3 | VUS |
| *LOXHD1* | c.3734G>A | p.(G1245D) | PM2, PP1_P | VUS |
| *LOXHD1* | c.5050G>A | p.(A1684T) | PM2, PP1_P, BP4_P | VUS |
| *MARVELD2* | c.1058dupT | p.(V354Sfs*5) | PVS1_VS, PM2, PP1_M | P |
| *MYH14* | c.1775G>A | p.(R592Q) | PM2_P, PP1_P | VUS |
| *MYO15A* | c.4778A>G | p.(E1593G) | PM1_M, PM2, PP1_P, PP3 | LP |
| *MYO15A* | c.6011C>T | p.(P2004L) | PM1_M, PM2_P, PP1_P, PM3_P | LP |
| *MYO15A* | c.8767C>T | p.(R2923X) | PVS1_VS, PM2_P, PP1_P, | LP |
| *MYO15A* | c.6551G>C | p.(C2184S) | PM1_M, PM2, PP1_P, PP3 | LP |
| *MYO15A* | c.8340G>A | p.(T2780T) (splicing) | PS1_S, PS3_S, PM2, PP1_P, PP3, | P |
| *MYO15A* | c.4216G>A | p.(E1406K) | PM1_M, PM2_P, PP1_P, PP3 | LP |

| | | | | |
|---|---|---|---|---|
| *MYO15A* | c.1196A>G | p.(Y399C) | BS1_P, PP1_P, PM3_P | VUS |
| *MYO15A* | c.6716A>C | p.(H2239P) | PM1_M, PM2, PP1_P, PP3 | LP |
| *MYO15A* | c.8065delT | p.(W2689Gfs*49) | PVS1_VS, PM2_P, PP1_P | LP |
| *MYO15A* | c.6518C>T | p.(S2173F) | PM1_M, PM2, PP1_P, PP3 | LP |
| *MYO15A* | c.6863C>T | p.(S2288L) | PM1_M, PM2_P, PP1_P | LP |
| *MYO15A* | c.4519C>T | p.(R1507X) | PVS1_VS, PM2, PP1_P | LP |
| *MYO15A* | c.6302T>C | p.(L2101P) | PM1_M, PM2_P, PP1_P, PP3 | LP |
| *MYO15A* | c.6634G>A | p.(E2212K) | PM1_M, PM2_P, PP1_P, PP3 | LP |
| *MYO15A* | c.9947A>G | p.(Q3316R) | PM1_M, PM2, PP1_P, PP3 | LP |
| *MYO7A* | c.1996C>T | p.(R666X) | PVS1_VS, PM2, PP1_P, | P |
| *MYO7A* | c.5101C>T | p.(R1701X) | PVS1_VS, PM2_P, PP1_P, | LP |
| *OTOF* | c.5813 +1G>A | | PVS1_VS, PM2, PP1_P | LP |
| *OTOF* | c.1747C>G | p.(P583A) | PM2_P, PP1_P, BP4_P, PM3_P | VUS |
| *OTOF* | c.1479_ 1481del | p.(K493_R494delinsN) | PM1_M, PM2, PM4_M, PP1_P, | LP |
| *OTOF* | c.1051C>T | p.(Q351X) | PVS1_VS, PM2, PP1_P | LP |
| *OTOG* | c.6982T>A | p.(S2328T) | PM2_P, PP1_P | VUS |
| *OTOG* | c.8513G>A | p.(R2838H) | PM2_P, PP1_P | VUS |
| *SLC12A2* | c.2935G>A | p.(E979K) | PM2, PM1, PP1_P | VUS |
| *SLC26A4* | c.2089 +1G>A | | PVS1_VS, PM2_P, PP1_P | LP |
| *SLC26A4* | c.1225C>T | p.(R409C) | PS1_S, PM1_M, PM2_P, PM5_M, PP1_P, PP3 | P |
| *TMC1* | c.1622T>A | p.(I541N) | PM2, PP1_P | VUS |
| *TRRAP* | c.1156G>A | p.(V386I) | PM2, PP1_P | VUS |

ACMG criteria* _VS = very strong, _S = strong, _M = moderate, and _P = supporting. Verdict*, P = pathogenic, LP = likely pathogenic, VUS = variant of uncertain significance, LB = likely benign, B = benign.

**Supplementary Table 3: Topmed, gnomAD, and CADD score of Candidate Gene Variants**

| Gene | NM numbers | Nucleotide change | Protein change | Rs-number | Affected families | gnomAD _AF | gnomAD_ AFR_AF | TOPMed | CADD_ Score |
|---|---|---|---|---|---|---|---|---|---|
| *INPP4B* | NM_003866.3 | c.1848G>C | p.(Q616H) | rs147919355 | 1 | 1.44E-04 | 2.16E-03 | 5.97E-04 | 23.7 |
| *INPP4B* | NM_003866.3 | c.1271T>C | p.(I424T) | rs747224392 | 1 | - | - | 7.18E-05 | 23.4 |
| *CCDC141* | NM_173648.3 | c.704A>G | p.(D235G) | rs1029313097 | 1 | 1.34E-05 | 2.91E-04 | 1.10E-04 | 24.6 |
| *CCDC141* | NM_173648.3 | c.202G>A | p.(E68K) | rs540836199 | 1 | 2.11E-04 | 3.75E-03 | 1.28E-03 | 25.6 |
| *PAX8* | NM_013952.3 | c.968C>G | p.(P323R) | rs1573435665 | 1 | - | - | 1.13E-5 | 14.55 |
| *MYO19* | NM_001163735.1 | c.2464- 8T>C | | rs183461441 | 1 | 1.31E-04 | 2.03E-03 | 7.06E-04 | 14.91 |
| *MYO19* | NM_001163735.1 | c.949G>T | p.(A317S) | rs199866785 | 1 | 1.41E-04 | 2.14E-03 | 7.10E-04 | 1.006 |
| *DNAH11* | NM_001277115.1 | c.11232C>G | p.(I3744M) | rs201120788 | 1 | 5.65E-05 | 5.18E-04 | 2.83E-04 | 0.001 |
| *DNAH11* | NM_001277115.1 | c.12969G>C | p.(Q4323H) | rs191802172 | 1 | 9.23E-05 | 1.42E-03 | 7.97E-04 | 22.6 |
| *SOX9* | NM_000346.3 | c.432-3C>A | | rs1033320617 | 1 | - | - | - | 22.2 |
| *POTEI* | NM_001277406.2 | c.1676G>C | p.(G559A) | rs1254207451 | 1 | - | - | 1.23E-3 | 0.065 |
| *POTEI* | NM_001371926.1 | c.409C>T | p.(R137X) | rs536831847 | 1 | 4.55E-6 | 7.44E-5 | 1.51E-5 | 2.707 |

Rs-number = Reference number, genotype, gnomAD_AF = global population allele frequency on the Genome Aggregation Database (gnomAD), gnomAD_AFR_AF = African population allele frequency on the Genome Aggregation Database (gnomAD),

## Supplementary Table 4: *In silico* evaluations of splice site variants identified in novel HI gene

<table>
<tr><td></td><td colspan="2">SOX9: c.432-3C>A (Fam 45)</td><td colspan="2">MYO19: c.2464-8T>C (Fam 40)</td></tr>
<tr><td>Tool</td><td>Prediction</td><td>Summary</td><td>Prediction</td><td>Summary</td></tr>
<tr><td>Mutation taster</td><td>Deleterious</td><td>Alteration within used splice site, likely to disturb normal splicing</td><td>Benign</td><td>The variant may not influence splicing</td></tr>
<tr><td rowspan="4">Splice AI</td><td rowspan="4">-</td><td>Acceptor Loss (0.02)</td><td rowspan="4">-</td><td>Acceptor Loss (0.16)</td></tr>
<tr><td>Donor Loss (0.00)</td><td>Donor Loss (0.00)</td></tr>
<tr><td>Acceptor Gain (0.18)</td><td>Acceptor Gain (0.00)</td></tr>
<tr><td>Donor Gain (0.00)</td><td>Donor Gain (0.00)</td></tr>
<tr><td>Human splice site finder</td><td>Broken WT Acceptor Site</td><td>Alteration of the WT Acceptor site, most probably affecting splicing</td><td>-</td><td>No significant impact on splicing signals</td></tr>
<tr><td rowspan="3">*Splice site prediction by neural network</td><td rowspan="3">Sequence: atttatttatttatttttaa gaaaagttatgagctgtggtt<br><br>Splice site prediction score = 0.66</td><td rowspan="3">The splice site prediction score is higher than the donor and acceptor score cutoffs</td><td>Sequence:<br>aatatccctttatagcttttagatttatctagccctcttagc<br><br>Splice site prediction score = 0.56</td><td>The splice site prediction score is higher than the donor and acceptor score cutoffs</td></tr>
<tr><td>Sequence:<br>tatagctttagatttatctagccctcttagcagaaaatg ga<br><br>Splice site prediction score = 0.28</td><td>The splice site prediction score is lower than the donor and acceptor score cutoffs</td></tr>
<tr><td>Sequence:<br>tttatctagccctcttagcagaaaatggaaatcaaaact at<br><br>Splice site prediction score = 0.57</td><td>The splice site prediction score is higher than the donor and acceptor score cutoffs</td></tr>
</table>

* A default donor and acceptor score cutoff 0.4 was used. This score corresponds to 93.2% site recognition, 5.2% false positive sites, and 0.84 correlation coefficient (CC) for human 5` splice site prediction, and 83.8% site recognition, 3.1% false positive sites, and 0.81 correlation coefficient (CC) for human 3` splice site prediction [21]. Exon/intron boundaries are shown in bold fonts.

## Supplementary Table 5: Variants found in unresolved families, without novel candidate genes.

| Family ID | Gene | nucleotide | Protein | Reference number | Note |
|---|---|---|---|---|---|
| Fam48 | *GJB2* | c.427C>T | p.(R143W) | rs80338948 | Variant did not segregate |
| Fam51 | *RPL5* | c.319C>T | p.(R107C) | rs777933769 | Incomplete segregation since phenotype and genotype data is unavailable for father |
| | *PIGT* | c.515G>A | p.(R172H) | rs750403754 | |
| Fam52 | *NCL* | c.2065G>A | p.(G689S) | | |
| | | c.188A>G | p.(K63R) | | |
| | *DACT2* | c.796-12C>T | | | The variants did not appear to be deleterious |
| | | c.1021T>G | | | |
| Fam4 | *BCORL1* | c.4079_ 4081del | p.(D1360_ L1361delinsV) | rs781578120 | The genotype does not corelate to clinical expression (non-syndromic HI). No strong evidence was found to support it as a novel gene |
| | *MYO15A* | c.6863C>T | p.(S2288L) | rs886052676 | A second *MYO15A* variant that could explain HI was not identified. |

## Supplementary Table 6: Allele specific primers for amplifying and sequencing *MYO19* and *DNAH11* regions with the variants of interest.

| Primer name | Sequence (5'->3') |
|---|---|
| DNAH11-Ex36-F | TGGTATCTTGGAATCAGCCATGGT |
| DNAH11-Ex36-R | GTACTGTGTACACTCCTCAGGTGC |
| DNAH11-Ex4-F | GCAAACCCATTAGCAAACAGAGA |
| DNAH11-Ex4-R | GCTGTGTCTCTCTACCAAGCA |
| DNAH11-ex45-F | AAGTGAACACCACCGTTACAA |
| DNAH11-ex45-R | TGGGCAATTTTGTCAGCCCA |
| MYO19-Ex16-F | GCCCTCAACTGCGTATTCCT |
| MYO19-Ex16-R | CTCTGGGCTGTCTTCACACT |
| MYO19-Ex26-F | CCTCAGTCTCATTCCCGCTG |
| MYO19-Ex26-R | AAGGCCTAAGCACAGTTGCC |

## Supplementary References


1. Kolla L, *et al.* Characterization of the development of the mouse cochlear epithelium at the single cell level. *Nat Commun* **11**, 1-16 (2020) doi:10.1038/s41467-020-16113-y.

2. Brunskill EW, *et al.* A gene expression atlas of early craniofacial development. *Dev Biol* **391**, 133-146 (2014) doi:10.1016/j.ydbio.2014.04.016.

3. Ebermann I, Lopez I, Bitner-Glindzicz M, Brown C, Koenekoop RK, Bolz HJ. Deafblindness in French Canadians from Quebec: a predominant founder mutation in the USH1C gene provides the first genetic link with the Acadian population. *Genome Biol* **8**, R47 (2007) doi:10.1186/gb-2007-8-4-r47.

4. Bujakowska KM, *et al.* Targeted exon sequencing in Usher syndrome type I. *Invest Ophthalmol Vis Sci* **55**, 8488-8496 (2014) doi:10.1167/iovs.14-15169.

5. Zhao L, *et al.* Next-generation sequencing-based molecular diagnosis of 82 retinitis pigmentosa probands from Northern Ireland. *Human genetics* **134**, 217-230 (2015) doi:10.1007/s00439-014-1512-7.

6. Zhang F, Xu L, Xiao Y, Li J, Bai X, Wang H. Three MYO15A Mutations Identified in One Chinese Family with Autosomal Recessive Nonsyndromic Hearing Loss. *Neural plasticity* **2018**, 5898025 (2018) doi:10.1155/2018/5898025.

7. Sarmadi A, Nasrniya S, Narrei S, Nouri Z, Abtahi H, Tabatabaiefar MA. Whole exome sequencing identifies novel compound heterozygous pathogenic variants in the MYO15A gene leading to autosomal recessive non-syndromic hearing loss. *Mol Biol Rep* **47**, 5355-5364 (2020) doi:10.1007/s11033-020-05618-w.

8. Jiang Y, *et al.* Mutation Spectrum of Common Deafness-Causing Genes in Patients with Non-Syndromic Deafness in the Xiamen Area, China. *PLoS One* **10**, e0135088 (2015) doi:10.1371/journal.pone.0135088.

9. Yuan Y, *et al.* Molecular epidemiology and functional assessment of novel allelic variants of SLC26A4 in non-syndromic hearing loss patients with enlarged vestibular aqueduct in China. *PLoS One* **7**, e49984 (2012) doi:10.1371/journal.pone.0049984.

10. Chai Y, *et al.* Molecular etiology of hearing impairment associated with nonsyndromic enlarged vestibular aqueduct in East China. *American journal of medical genetics Part A* **161A**, 2226-2233 (2013) doi:10.1002/ajmg.a.36068.

11. Mori K, Miyanohara I, Moteki H, Nishio S-y, Kurono Y, Usami S-i. Novel Mutations in GRXCR1 at DFNB25 lead to progressive hearing loss and dizziness. *Annals of Otology, Rhinology & Laryngology* **124**, 129S-134S (2015)

12. Bonnet C, *et al.* Complete exon sequencing of all known Usher syndrome genes greatly improves molecular diagnosis. *Orphanet J Rare Dis* **6**, 21 (2011) doi:10.1186/1750-1172-6-21.

13. Ouyang XM, *et al.* Characterization of Usher syndrome type I gene mutations in an Usher syndrome patient population. *Human genetics* **116**, 292-299 (2005) doi:10.1007/s00439-004-1227-2.

14. Khateb S, *et al.* The Genetics of Usher Syndrome in the Israeli and Palestinian Populations. *Invest Ophthalmol Vis Sci* **57**, 3154-3154 (2016)

15. Stabej PL, *et al.* Comprehensive sequence analysis of nine Usher syndrome genes in the UK National Collaborative Usher Study. *Journal of Medical Genetics* **49**, 27-36 (2012) doi:10.1136/jmedgenet-2011-100468.

16. Lee CG, Jang J, Jin HS. A novel missense mutation in the ACTG1 gene in a family with congenital autosomal dominant deafness: A case report. *Molecular medicine reports* **17**, 7611-7617 (2018) doi:10.3892/mmr.2018.8837.

17. Patel K, *et al.* A novel C-terminal CIB2 (calcium and integrin binding protein 2) mutation associated with non-syndromic hearing loss in a hispanic family. *PLoS One* **10**, e0133082 (2015)

18. Riazuddin S, *et al.* Alterations of the CIB2 calcium- and integrin-binding protein cause Usher syndrome type 1J and nonsyndromic deafness DFNB48. *Nature genetics* **44**, 1265-1271 (2012) doi:10.1038/ng.2426.

19. Seco CZ, *et al.* Novel and recurrent CIB2 variants, associated with nonsyndromic deafness, do not affect calcium buffering and localization in hair cells. *European Journal of Human Genetics* **24**, 542-549 (2016)

20. Mutai H, *et al.* Variants encoding a restricted carboxy-terminal domain of SLC12A2 cause hereditary hearing loss in humans. *PLoS Genet* **16**, e1008643 (2020) doi:10.1371/journal.pgen.1008643.

21. Reese MG, Eeckman FH, Kulp D, Haussler D. Improved splice site detection in Genie. *J Comput Biol* **4**, 311-323 (1997) doi:10.1089/cmb.1997.4.311.
