# Supplementary material for: Exome sequencing of families from Ghana reveals known and candidate hearing impairment genes
Source: Commun Biol. 2022 Apr 19;5:369. doi: 10.1038/s42003-022-03326-8 (PMC9019055; doi:10.1038/s42003-022-03326-8)
Supplement: Supplementary file 3 — Description of Additional Supplementary Files [file 42003_2022_3326_MOESM3_ESM.pdf]

Description of Additional Supplementary Files

File Name: Supplementary Data 1

Description: Data on variant classification and expression of novel candidate genes in the cochlea and utricle during mouse development.

File Name: Supplementary Data 2

Description: Data used for descriptive statistics of socio-demographic characterization of participants, pedigrees analysis, and differential quantitative average expression of novel candidate genes in craniofacial tissues during mouse development.
