# Supplementary material for: Exome sequencing of families from Ghana reveals known and candidate hearing impairment genes
Source: Commun Biol. 2022 Apr 19;5:369. doi: 10.1038/s42003-022-03326-8 (PMC9019055; doi:10.1038/s42003-022-03326-8)
Supplement: Supplementary file 6 — Reporting Summary [file 42003_2022_3326_MOESM6_ESM.pdf]

nature portfolio

Corresponding author(s): Ambroise Wonkam, and Suzanne M. Leal

Last updated by author(s): Nov 12, 2021

Reporting Summary

Nature Portfolio wishes to improve the reproducibility of the work that we publish. This form provides structure for consistency and transparency in reporting. For further information on Nature Portfolio policies, see our Editorial Policies and the Editorial Policy Checklist.

Statistics

For all statistical analyses, confirm that the following items are present in the figure legend, table legend, main text, or Methods section.

n/a	Confirmed	
☐	☒	The exact sample size (n) for each experimental group/condition, given as a discrete number and unit of measurement
☒	☐	A statement on whether measurements were taken from distinct samples or whether the same sample was measured repeatedly
☐	☒	The statistical test(s) used AND whether they are one- or two-sided<br>*Only common tests should be described solely by name; describe more complex techniques in the Methods section.*
☒	☐	A description of all covariates tested
☐	☒	A description of any assumptions or corrections, such as tests of normality and adjustment for multiple comparisons
☒	☐	A full description of the statistical parameters including central tendency (e.g. means) or other basic estimates (e.g. regression coefficient) AND variation (e.g. standard deviation) or associated estimates of uncertainty (e.g. confidence intervals)
☐	☒	For null hypothesis testing, the test statistic (e.g. F, t, r) with confidence intervals, effect sizes, degrees of freedom and P value noted<br>*Give P values as exact values whenever suitable.*
☒	☐	For Bayesian analysis, information on the choice of priors and Markov chain Monte Carlo settings
☒	☐	For hierarchical and complex designs, identification of the appropriate level for tests and full reporting of outcomes
☒	☐	Estimates of effect sizes (e.g. Cohen's d, Pearson's r), indicating how they were calculated

Our web collection on statistics for biologists contains articles on many of the points above.

Software and code

Policy information about availability of computer code

Data collection	1- The exome library for 129 samples (batch one) was prepared using the Nextera Rapid Capture Exome kit (Illumina, San Diego, CA)<br>2- Libraries were sequenced on an Illumina HiSeq 2500 sequencer (Illumina, San Diego, CA)<br>3-The exome library preparation of the rest of the samples (n = 51, batch two) was performed using SureSelect V4+UTR 71Mb All Exon Capture Kit (Agilent Technologies, Inc., Santa Clara, CA, USA)<br>4- The Illumina DRAGEN Germline Pipeline v3.2.8 was used to align the sequence reads to the human reference genome (hg19) and variants were jointly called using the Genome Analysis Toolkit (GATKv4.1.7) software package<br>5- Variant qualities were assessed by variant quality score recalibration (VQSR) using the ApplyVQSR function of GATK.
Data analysis	Code availability statements<br>Any previously unreported custom computer code or algorithm used to generate results that are reported in the paper and central to its main claims are available from corresponding author upon request.<br><br>Data analysis methods:<br>1- An in-house pipeline that uses ANNOVAR, dbNSFP, and dbscSNV was used to annotate and filter single-nucleotide, and insertion/deletion (indels) variants.<br>2- Filtering was performed using Genome Aggregation Database (gnomAD) population-specific minor allele frequency (MAF) of <0.005 [for AR and X-linked (XL)] and <0.0005 for autosomal dominant (AD) with variants meeting these criteria being further ranked based on the bioinformatics prediction scores from SIFT, polymorphism phenotyping v2 (PolyPhen-2), MutationTaster, combined annotation dependent depletion (CADD); deleterious annotation of genetic variants using neural networks (DANN); and Genomic Evolutionary Rate Profiling (GERP+ +).<br>3-Information from the Hereditary Hearing Loss Homepage (HHL) , Online Mendelian Inheritance in Man (OMIM), Human Phenotype Ontology (HPO), and ClinVar databases and deafness animal models were also used to prioritize identified variants. The MAFs of variants were further evaluated using the TOPMed Bravo database.<br>4- In families for which candidate variant(s) were not identified exome sequence data were also analysed for copy number variation (CNV)

using CoNIFER.
5- These variants were assessed for their clinical significance based on the ACMG-AMP classification, considering CADD scores, and their allele frequencies in gnomAD and TOPMed databases (Tables S1 and S2).
6- To study the expression during mouse craniofacial development, series GSE55966 from the Gene Expression Omnibus (GEO) database was evaluated. Craniofacial gene expression data were presented as a set of FPKM (Fragments Per Kilobase Million) values for 13 different tissue/stage pairs which were converted to TPM (Transcripts Per Kilobase Million) values for our analysis.
7- We also studied expression levels during mouse inner ear development in previously generated datasets present in the Shared Harvard Inner-Ear Laboratory Database (SHIELD). The expression data were produced by Affymetrix Mouse 420 v.2 GeneChips and subset into expression in spiral ganglion neurons and vestibular ganglion neuron.

For manuscripts utilizing custom algorithms or software that are central to the research but not yet described in published literature, software must be made available to editors and reviewers. We strongly encourage code deposition in a community repository (e.g. GitHub). See the Nature Portfolio guidelines for submitting code & software for further information.

Data

Policy information about availability of data

All manuscripts must include a data availability statement. This statement should provide the following information, where applicable:

- Accession codes, unique identifiers, or web links for publicly available datasets
- A description of any restrictions on data availability
- For clinical datasets or third party data, please ensure that the statement adheres to our policy

Data Availability Statement

The data that support the findings of this study are not openly available due to sensitivity i.e. human data, and are available from the corresponding author upon reasonable request; all data's are located in a controlled access repository , at the University of Cape Town, South Africa

Field-specific reporting

Please select the one below that is the best fit for your research. If you are not sure, read the appropriate sections before making your selection.

☒ Life sciences ☐ Behavioural & social sciences ☐ Ecological, evolutionary & environmental sciences

For a reference copy of the document with all sections, see nature.com/documents/nr-reporting-summary-flat.pdf

Life sciences study design

All studies must disclose on these points even when the disclosure is negative.

Sample size

The sample size calculation is not applicable to this study

Data exclusions

Not applicable

Replication

1- Replication human studied for Novel Candidate Genes: we explore our data base for additional families (with positive outcome for two genes in our Ghanean cohort), as well as submitted the candidate to the matchmaker exchange home page to seek for possible additional families with international investigators (https://www.matchmakerexchange.org/)
2- Replication in independent cohort: We found variants in or near the 7 novel candidate genes in possible association to hearing impairment as well (+/- 100kb), in the latest genome-wide association data browser from the FinnGen research project (Release 5; 218,792 individuals; https://r5.finngen.fi/), which aims to study genetic variation associations with various traits in the isolated population of Finland. This includes variants in or near CCDC141 (rs144697379; p=2.6 x 10-5; intronic), DNAH11 (rs2965393; p=4.5 x 10-4; intronic), PAX8 [rs115708270; p=3.6 x 10-4; upstream] and SOX9 (rs16977126; p=5.7x10-5; downstream) for sensorineural hearing loss, and in MYO19 [rs143245472;p=6.6 x 10-6; upstream], INPP4B [rs184880581; p=2.6 x 10-5; intronic] and POTEI [rs1337496945; p= 5.6 x 10-4; downstream] for sudden idiopathic hearing loss.

Randomization

Not Applicable

Blinding

The blinding was not relevant to this study design: pedigrees analysis, and variants segregation with early onset hearing impairment.

Reporting for specific materials, systems and methods

We require information from authors about some types of materials, experimental systems and methods used in many studies. Here, indicate whether each material, system or method listed is relevant to your study. If you are not sure if a list item applies to your research, read the appropriate section before selecting a response.

Materials & experimental systems

n/a	Involved in the study
☒	☐ Antibodies
☒	☐ Eukaryotic cell lines
☒	☐ Palaeontology and archaeology
☐	☒ Animals and other organisms
☐	☒ Human research participants
☒	☐ Clinical data
☒	☐ Dual use research of concern

Methods

n/a	Involved in the study
☒	☐ ChIP-seq
☒	☐ Flow cytometry
☒	☐ MRI-based neuroimaging

Animals and other organisms

Policy information about studies involving animals; ARRIVE guidelines recommended for reporting animal research

Laboratory animals

Expression of candidate genes in the developing and adult mouse inner ear

Various publicly available RNA sequencing and microarray datasets were used for an in silico investigation of the expression of CCDC141, DNAH11, INPP4B, MYO19, PAX8, and SOX9 in the developing and adult mouse inner ear. To study the expression during mouse craniofacial development, series GSE55966 from the Gene Expression Omnibus (GEO) database was evaluated. This dataset includes RNA sequence data of CD1 mouse embryos at three stages: E8.5, E9.5, and E10.5 33. Craniofacial gene expression data were presented as a set of FPKM (Fragments Per Kilobase Million) values for 13 different tissue/stage pairswhich were converted to TPM (Transcripts Per Kilobase Million) values for our analysis.
We also studied expression levels during mouse inner ear development in previously generated datasets present in the Shared Harvard Inner-Ear Laboratory Database (SHIELD). The first dataset detailed expression over four developmental stages: E16, P0, P4, and P735. Data were obtained from the cochlea and utricles of mice that expressed EGFP under the Pou4f3 promoter. Fluorescence-Activated Cell Sorting (FACS) was used to separate cells into hair cells (GFP+) and surrounding cells (GFP-) prior to RNA extraction 35. To supplement these data, a second dataset was downloaded, which contained expression data for six developmental stages: E12, E13, E16, P0, P6, and P15. The expression data were produced by Affymetrix Mouse 420 v.2 GeneChips and subset into expression in spiral ganglion neurons and vestibular ganglion neurons.
Lastly, the gene Expression Analysis Resource (gEAR) suite was used to visualize expression in cells of the cochlear epithelium during mouse development. gEAR includes single-cell RNA sequence data obtained from CD1 mouse embryos at four developmental stages: E14, E16, P1, and P7 37. CCDC141, DNAH11, INPP4B, MYO19, and SOX9 expression was also grouped based on cell groups in four overarching classes: developing supporting cells, developing prosensory cells, developing sensory cells, and developing greater epithelial ridge cells of which each were further divided into subclasses .

Wild animals

Not applicable

Field-collected samples

Not applicable

Ethics oversight

Not applicable

Note that full information on the approval of the study protocol must also be provided in the manuscript.

Human research participants

Policy information about studies involving human research participants

Population characteristics

Patient recruitment procedures were previously described as follows: the probands of Ghanaian families segregating HI were identified through 9 schools for the deaf across the country (Figure 1), and additional family members were recruited thereafter. Families with HI were also identified and recruited through our community engagement activities 13 (Figure 1). A total of 5ml of peripheral blood was obtained from each participant and genomic DNA (gDNA) was extracted from the samples using the QIAamp DNA Blood Maxi Kit ® (Qiagen, USA).

Recruitment

Medical records of all our participants were reviewed by a medical geneticist, and an ear, nose, and throat specialist. Detailed personal and family histories were obtained through a rigorous clinical interview. A structured questionnaire was used to interview each participant to rule out potential environmental causes of HI. The studied families had at least two family members affected with HI that followed a mendelian mode of inheritance. For those participants that were ascertained from schools for the deaf, before being admitted to the school they had undergone a systemic general and otological examination, that included pure tone audiometry.

Ethics oversight

We observed and adhered strictly to the guiding principles of the Declaration of Helsinki. Ethical approvals were obtained from Noguchi Memorial Institute for Medical Research Institutional Review Board (IRB) (NMIMR-IRB CPN 006/16-17), the University of Cape Town, Faculty of Health Sciences' Human Research Ethics Committee (HREC 104/2018), and the IRB of Columbia University (IRB-AAAS2343). The study was clearly explained to each participant in the language in which they are fluent and informed consent was signed prior to participation. Parents/guardians provided signed consent for their children who were <18 years of age. In addition, assent was obtained for children older than seven years of age.

Note that full information on the approval of the study protocol must also be provided in the manuscript.
