# Supplementary material for: Exome sequencing of families from Ghana reveals known and candidate hearing impairment genes
Source: Commun Biol. 2022 Apr 19;5:369. doi: 10.1038/s42003-022-03326-8 (PMC9019055; doi:10.1038/s42003-022-03326-8)
Supplement: Supplementary file 1 — Peer Review File [file 42003_2022_3326_MOESM1_ESM.pdf]

nature portfolio

Peer Review File

Exome sequencing of families from Ghana reveals known and candidate hearing impairment genes

Open Access This file is licensed under a Creative Commons Attribution 4.0 International License, which permits use, sharing, adaptation, distribution and reproduction in any medium or format, as long as you give appropriate credit to the original author(s) and the source, provide a link to the Creative Commons license, and indicate if changes were made. In the cases where the authors are anonymous, such as is the case for the reports of anonymous peer reviewers, author attribution should be to 'Anonymous Referee' followed by a clear attribution to the source work. The images or other third party material in this file are included in the article's Creative Commons license, unless indicated otherwise in a credit line to the material. If material is not included in the article's Creative Commons license and your intended use is not permitted by statutory regulation or exceeds the permitted use, you will need to obtain permission directly from the copyright holder. To view a copy of this license, visit http://creativecommons.org/licenses/by/4.0/.

This manuscript has been previously reviewed at another Nature Portfolio journal. This document only contains reviewer comments and rebuttal letters for versions considered at Communications Biology.

REVIEWERS' COMMENTS:

Reviewer #1 (Remarks to the Author):

Hearing loss is a sensory disorder which affects quality of life of the patients. It is often associated with genetical disorder. In this manuscript, Wonkam et al., have investigated whole exome sequencing analysis to identify genes involved in hearing impairment in 51 families from Ghana. The authors identified known genes involving hearing impairment and novel genes which might be associated with deafness genetically. This finding is interesting and informative. As the reviewer 2 pointed out data regarding latter genes are preliminary and descriptive. Especially, functional analysis to support the role of those genes in hearing loss are not presented. However, this report would stimulate the field for further study. As the authors replied, I feel that the functional analysis ranging from in vitro study to mice experiments is out of the scope of this study. To improve the manuscript, is it possible to examine a kind of gene network analysis? It would be great if the authors show potential connection of newly identified genes with known causative genes involved in hearing loss.
